# High dimensional model representation of log likelihood ratio: binary classification with SNP data

**DOI:** 10.1186/s12920-020-00774-1

**Published:** 2020-09-21

**Authors:** Ali Foroughi pour, Maciej Pietrzak, Lara E. Sucheston-Campbell, Ezgi Karaesmen, Lori A. Dalton, Grzegorz A. Rempała

**Affiliations:** 1 Department of Electrical and Computer Engineering, The Ohio State University, 2015 Neil Ave, Columbus, 43210 OH USA; 2 Department of Mathematics, The Ohio State University, 231 West 18th Ave, Columbus, 43210 OH USA; 3 Mathematical Biosciences Institute, 1735 Neil Ave, Columbus, 43210 OH USA; 4 College of Public Health, The Ohio State University, 1841 Neil Ave, Columbus, 43210 OH USA; 5 Department of Biomedical Informatics, The Ohio State University, 1585 Neil Ave, Columbus, 43210 OH USA; 6 College of Pharmacy, The Ohio State University, 500 West 12th Ave, Columbus, 43210 OH USA

**Keywords:** Single nucleotide polymorphism, Binary classification, High dimensional model representation, Pairwise SNP interactions, Log likelihood ratio

## Abstract

**Background:**

Developing binary classification rules based on SNP observations has been a major challenge for many modern bioinformatics applications, e.g., predicting risk of future disease events in complex conditions such as cancer. Small-sample, high-dimensional nature of SNP data, weak effect of each SNP on the outcome, and highly non-linear SNP interactions are several key factors complicating the analysis. Additionally, SNPs take a finite number of values which may be best understood as ordinal or categorical variables, but are treated as continuous ones by many algorithms.

**Methods:**

We use the theory of high dimensional model representation (HDMR) to build appropriate low dimensional glass-box models, allowing us to account for the effects of feature interactions. We compute the second order HDMR expansion of the log-likelihood ratio to account for the effects of single SNPs and their pairwise interactions. We propose a regression based approach, called *linear approximation for block second order HDMR expansion of categorical observations* (LABS-HDMR-CO), to approximate the HDMR coefficients. We show how HDMR can be used to detect pairwise SNP interactions, and propose the *fixed pattern test* (FPT) to identify statistically significant pairwise interactions.

**Results:**

We apply LABS-HDMR-CO and FPT to synthetically generated HAPGEN2 data as well as to two GWAS cancer datasets. In these examples LABS-HDMR-CO enjoys superior accuracy compared with several algorithms used for SNP classification, while also taking pairwise interactions into account. FPT declares very few significant interactions in the small sample GWAS datasets when bounding false discovery rate (FDR) by 5%, due to the large number of tests performed. On the other hand, LABS-HDMR-CO utilizes a large number of SNP pairs to improve its prediction accuracy. In the larger HAPGEN2 dataset FTP declares a larger portion of SNP pairs used by LABS-HDMR-CO as significant.

**Conclusion:**

LABS-HDMR-CO and FPT are interesting methods to design prediction rules and detect pairwise feature interactions for SNP data. Reliably detecting pairwise SNP interactions and taking advantage of potential interactions to improve prediction accuracy are two different objectives addressed by these methods. While the large number of potential SNP interactions may result in low power of detection, potentially interacting SNP pairs, of which many might be false alarms, can still be used to improve prediction accuracy.

## Background

Many modern bioinformatics applications utilize data analysis methods originally developed and studied in the fields of statistics, signal processing, and machine learning. In particular, in many cases, the application can be formulated as a classification or regression problem. Data encountered in bioinformatics is typically “small-sample high-dimensional”, a challenge not encountered in many classical statistics problems and machine learning applications. For instance, to predict the risk of a person being diagnosed with a specific complex disease in the future, or to predict the effect of a treatment, e.g., for targeted therapy, one may collect several hundred thousand single nucleotide polymorphisms (SNPs) in a sample of several hundred or a few thousand patients with known labels. Although current “omics” data provide a deluge of information per sample point, research being restricted to small sample sizes impedes reliable analysis. While being a small-sample high-dimensional problem is typical of many bioinformatics applications, it seems to be more pronounced when analyzing SNPs as (a) current technologies measure hundreds of thousands of SNPs, and (b) sample size can easily be much smaller than the number of disease associated SNPs. Furthermore, many molecular features, and in particular SNPs, can be weak markers, meaning each individual feature alone cannot reliably predict the disease outcome, and a large collection of features need to be considered together to obtain reliable predictions. Additionally, biological features are typically heavily dependent, for instance due to linkage disequilibrium (LD), and have complex interactions, which may exacerbate the difficulties in developing accurate prediction rules. Finally, note that interpretability is an important aspect in biological research. Not only do we look for biological markers and prediction rules with high accuracy, but also require a glass-box model that can explain “how” and “why” the prediction rule has come to a specific decision. For example, that a specific mutation increases cancer risk by some amount, or presence of a combination of mutations is an indicator of high risk.

To that end, many pipelines implement a first phase of feature selection to reduce dimensionality, improve replicability, and increase prediction accuracy. It has been shown in many studies that such approach in indeed valuable and useful in practical applications [1–4]. Additionally, penalized methods, such as those using LASSO or elastic net penalties, are widely used. Although feature selection as a means of dimensionality reduction is helpful, it is not always sufficient. For instance, the number of disease associated SNPs passing the selection stage can still be too large compared with sample size.

Due to the large number of biological markers, their complex interactions, the need for interpretability, and the relative lack of large datasets as compared with other machine learning applications, it is typically desired to use low dimensional generative models. The idea is that although the “optimal” rule can be highly complex, it can be well approximated by a low dimensional model, and a proper low dimensional family, for instance generalized linear models (GLMs) with logit or probit links, is large enough to contain a point close to the best low dimensional representation. Thereby, such approximation will avoid over-fitting, improving prediction reliability and accuracy.

SNPs are among the most challenging biological features to analyze. Indeed a single mutation in the deoxyribonucleic acid (DNA) might not greatly impact the risk of a complex disease. Therefore, it is reasonable to assume mutations are weak markers that should be jointly studied to arrive at a reliable decision rule. For instance, GLMs with logit link that take dosage data, i.e., the number of minor alleles at each SNP, are a very popular, if not the most popular, method for binary classification given SNP data. Refer to [3–8] for such examples. Additionally, GLMs can be used with sparsity inducing penalties, such as elastic net, and can include product terms of two SNPs to account for their interactions. However, in many cases it is not possible to use all strong pairwise interactions in the data for classification. For instance, given 1000 disease associated SNPs, there are about 500,000 SNP pairs that can be considered in the classification rule. In absence of biological information or given a set of known interacting SNP pairs, it is typically not computationally feasible to consider all possible pairwise interactions and use sparsity inducing penalties such as LASSO and elastic net. Furthermore, such formulations may enforce a linear risk function for many SNPs, implying that a SNP with two minor alleles should induce a risk twice of that SNP having one minor allele, which might not be a valid assumption. For example, for a recessive SNP we may have that only presence of two minor alleles increases the risk, while one minor allele has no effect on the risk. We would like to emphasize that we found little discussion studying how valid the logit link assumption with linear additive risks is in practice.

Support vector machines (SVM), random forests, *k* nearest neighbors (*k*NN), and naive Bayes are other methods used for binary classification of SNP data [3, 4, 6, 7]; however, they are not as popular as GLMs, even though in many cases they are suggested to outperform GLMs. Note that many of these methods, such as SVMs and *k*NN, require a notion of distance, meaning they interpret the number of minor alleles as real numbers, although it might be more suitable to treat dosage data as ordinal variables. Treating dosage data as real-valued variables, although they take a finite number of values, whether understood as ordinal or categorical variables, may be a reason why many off-the-shelf methods, despite outperforming GLMs, do not perform adequately on SNP data.

Here we use the theory of high dimensional model representation (HDMR) to find the “best” second order approximation of the log likelihood ratio, and solve it for the case of categorical observations, which we believe is a better approach to model SNP data than treating dosage values as real numbers. By considering a second order expansion we can account for the effect of single SNPs and pairwise SNP interactions, where by SNP interactions we understand the non-zero terms in the second order HDMR expansion of the log likelihood ratio borrowing from two SNPs which is explained in more detail the “Methods” section. Additionally, we propose linear approximations based on the objectives studied in compressed sensing to approximate the second order HDMR expansion. We use the Sobol indices, an extension of the *R*^2^ statistic that is closely connected to the HDMR expansion, to compute statistics indicating whether there is a significant interaction for a specific SNP pair value. We apply the developed method to a simulated data based on the HAPGEN2 [9] project, as well as lung and breast cancer datasets, showing the proposed methods enjoy higher classification accuracies as well as being able to efficiently detecting strong pairwise SNP interactions.

## Methods

Here we describe our classification methodology based on the High Dimensional Model Representation (HDMR) expansion, studied in detail in [10–13]. We first briefly review the general theory, how it applies to a binary classification problem, and how the categorical observations simply the process.

### High dimensional model representation

HDMR is a powerful tool to represent a function of a random vector based on marginal observations. HDMR provides us with a hierarchy of functions that describe how the interactions of variables affect the output. In particular, assuming output *Z* is a function of input random vector *X*=[*X*_1_,⋯,*X*_*D*_], i.e., *Z*=*f*(*X*), HDMR decomposes *f*(*X*) based on partial observations. Let *F*={1,⋯,*D*}. The HDMR expansion of *Z* is the collection of functions *f*_*u*_(*X*_*u*_) for all *u*⊆*F* such that

1 $$\begin{array}{*{20}l}  f_{u}(x_{u})=\underset{g_{u}(x_{u}) \in L^{2}(\mathbb{R}^{|u|}) }{argmin} \int \left(\sum\limits_{u} g(u)-f(X)\right)^{2} d\mu, \end{array} $$

under the condition that

2 $$\begin{array}{*{20}l}  \forall u \subseteq \{1,\cdots,D\}, \forall i \in u \int f_{u}(x_{u}) w(x) dx_{i} dx_{-u} =0. \end{array} $$

where for each $x \in \mathbb {R}^{D}$, *x*_*u*_ is the restriction of *x* to elements in *u*, *x*_−*u*_ is the restriction of *x* to elements not in *u*, and *μ* is the probability measure of random vector *X* described by probability density function (p.d.f.) *w* [12]. Note this condition is equivalent to a hierarchical orthogonality criterion of the following form [12]:

3 $$\begin{array}{*{20}l}  \forall v\subset u, \forall g_{v}: \int f_{u}(x_{u}) g_{v}(x_{v}) w(x) dx =0. \end{array} $$

Therefore, via the HDMR expansion we may write

4 $$\begin{array}{*{20}l}  f(X)=f_{0}+\sum\limits_{\substack{u \subseteq F \\ u \neq \phi}} f_{u}(X_{u}), \end{array} $$

where

5 $$\begin{array}{*{20}l}  f_{0}&=\int f(x) w(x) dx, \end{array} $$

6 $$\begin{array}{*{20}l}  f_{u}&=\int f(x) w(x_{-u}) dx_{-u}  \\ &- \sum\limits_{v \subset u} f_{v}(x_{v}) - \sum\limits_{v\neq u: v \cap u \neq \phi} \int f_{v}(x_{v}) w_{-u} dx_{-u}. \end{array} $$

Equation 6 suggests that in the general case of dependent variables a component function, *f*_*u*_(*x*_*u*_) depends on all other expansion terms that also have a non-empty intersection with *u*. However, assuming elements of *X* are independent, the last term of (6) equals zero and we may write

7 $$\begin{array}{*{20}l} f_{u}&=\int f(x) w(x_{-u}) dx_{-u} - \sum\limits_{v \subset u} f_{v}(x_{v}). \end{array} $$

While this greatly simplifies the process of computing the HDMR expansion, the independence assumptions is too strong for SNPs, as they can be heavily correlated. Observe that by considering sets *u* such that |*u*|≤*d* in (4) we arrive a the *d*^*th*^ order HDMR expansion of *Z*, which we hereafter denote by *E*_*d*_(*Z*|*X*).

### Second order HDMR for categorical observations

Now, additionally consider the case where *X* is a categorical random vector. In particular, for each *f*∈*F*, *X*_*f*_ is a categorical random variable with support *C*_*f*_. In other words, *C*_*f*_ is the collection of categories *X*_*f*_ may take. Now, assuming *X* is categorical, the domain of *f*_*u*_(*x*_*u*_) is the finite collection of all combinations in $C_{u}=\prod \nolimits _{f \in u} C_{f}$. Therefore, we may write

8 $$\begin{array}{*{20}l} f_{u}(x_{u})=\sum\limits_{c \in C_{u}} q^{c}_{u} 1_{X_{u}=c}, \end{array} $$

where 1_*q*_ is the indicator function of statement *q* being true. Additionally, for the case of second order HDMR expansion we have

$$\begin{array}{*{20}l} E\left(Z|X_{f}\right)&=\sum\limits_{c \in C_{f}} q_{f}^{c} 1_{X_{f}=c},  \\ E\left(Z|X_{f,f'}\right)&=\sum\limits_{c \in C_{f}} \sum\limits_{c' \in C_{f'}} q_{f,f'}^{c,c'} 1_{X_{f}=c,X_{f'}=c'},  \end{array} $$

for some $q_{f}^{c}, q_{f,f'}^{c,c'} \in \mathbb {R}$. Therefore, we can further simplify and write

9 $$\begin{array}{*{20}l}  &E_{2}(Z|X) =q_{0}+\sum\limits_{f \in F} \sum\limits_{c_{f} \in C_{f}} q^{c_{f}}_{f} \times 1_{x_{f}=c}  \\ &+ \sum\limits_{\substack{f_{i},f_{j} \in F \\ i< j}} \sum\limits_{\substack{c_{f_{i}} \in C_{f_{i}}\\c_{f_{j}} \in C_{f_{j}}}} q_{f_{i}f_{j}}^{c_{f_{i}} c_{f_{j}}} \times 1_{\left\{X_{i}=c_{f_{i}}, X_{j}=c_{f_{j}}\right\}}, \end{array} $$

for some $ q_{0},q^{c_{f}}_{f}, q_{f_{i}f_{j}}^{c_{f_{i}} c_{f_{j}}} \in \mathbb {R}$. Note instead of indicators $1_{x_{f}=c}$ and $1_{\left \{X_{i}=c_{f_{i}}, X_{j}=c_{f_{j}}\right \}}$, we can basically do a change of basis, and use a new set of indicators that are linearly independent and uniquely tell us the value of the categorical observation. More precisely, we may consider each $Z^{f}_{c}=1_{\left \{X_{f}=c\right \}}$ as a binary random variable, and directly write the HDMR expansion using $Z^{f}_{c}$’s. Therefore, without loss of generality, we may assume |*C*_*f*_|=2. Additionally, let $Q_{f}=\left \{q^{i}_{f}: i=1,\cdots,|C_{f}|-1 \right \}$ for be a collection of statements that uniquely determine the value of *X*_*f*_, although they might not necessarily be in the form of $1_{\{X_{f}=c\}}$. For example, suppose *C*_*f*_={0,1,2}. Instead of statements $1_{\left \{X_{f}=0\right \}}$ and $1_{\left \{X_{f}=1\right \}}$ to determine the value of *X*_*f*_, we can also use $1_{\left \{X_{f} \geq 1\right \}}$ and $1_{\left \{X_{f} =2 \right \}}$ to determine the category of *X*_*f*_. Note for both cases we have $1_{\left \{X_{f}=2\right \}}=1-1_{\left \{X_{f}=0\right \}}-1_{\left \{X_{f}=1\right \}}$ and $1_{\left \{X_{f}=1\right \}}=1_{\left \{X_{f} \geq 1\right \}} - 1_{\left \{X_{f} =2\right \}}$. Therefore, we can further simplify and write

10 $$\begin{array}{*{20}l}  E_{2}(L(X)|X)& = E(L(X))+\sum\limits_{f \in F} \sum\limits_{q \in Q_{f}} w^{q}_{f} q \\&+ \sum\limits_{\substack{f_{i},f_{j} \in F \\ i< j}} \sum\limits_{q_{i} \in Q_{f_{i}}} \sum\limits_{q_{j}\in Q_{f_{j}}} w^{q_{i} q_{j}}_{f_{i} f_{j}} q_{i} q_{j}. \end{array} $$

Here our goal is to analyze SNP data when they are reported in dosage, i.e., for each SNP *f* we report the number of minor alleles, and hence *C*_*f*_={0,1,2}. We hereafter mainly focus on this special case to outline the procedure for estimating coefficients $b^{q}_{f}$ and $b^{q_{i} q_{j}}_{f_{i} f_{j}}$ up to an affine transformation; however, the algorithms developed are more general. Note with little abuse of terminology, we use SNP to refer to the categorical dosage value, being the number of minor alleles. Our specific choice of *Q*_*f*_ is $1_{\left \{X_{f} \geq 1\right \}}$ and $1_{\left \{X_{f} =2 \right \}}$, which will be made clear later. Note a deeper discussion on the HDMR expansion using extended bases can be found in [11, 14, 15].

### HDMR expansion for binary classification

Here we describe how HDMR can be used for a binary classification problem. Consider a binary classification problem with class labels *y*=0,1 and feature index set *F*. Let *X* be a random unlabeled observation with true label *y*_*x*_. Given *S*, it is desired to design a decision rule that assigns a label, $\hat {y}_{x}$ to *X* so that $\hat {y}_{x}=y_{x}$ with high probability. Note given the full joint distribution of *X* and *y*_*x*_, one could have easily computed *P*(*y*_*x*_=1|*X*), or equivalently the log likelihood ratio *L*(*X*)= log(*P*(*y*_*x*_=1|*X*)/*P*(*y*_*x*_=0|*X*)), and use a decision rule $\hat {y}_{x}=1_{L(X)>T}$, where 1_*q*_ is the indicator function of statement *q* being correct, and *T* is a threshold.

However, the full joint distribution is typically not available, as is usually estimated given training sample, *S*. Alternatively, many models assume the classification rule belongs to a family of rules parametrized by *θ*, and aim to estimate *θ* given *S*. For example, a GLM using the famous logit link assumes $L(X)=\beta _{0}+ {\sum \nolimits }_{f \in F}\beta _{f} X_{f}$, where *X*_*f*_ is the value of *X* for feature *f*, and *θ* is the collection of *β*_0_ and *β*^*f*^’s. However, such model is insufficient for many applications where it is desired to account for pairwise SNP interactions, and is not easy to train using LASSO and elastic net penalties while accounting for pairwise interactions by adding terms of the form $\phantom {\dot {i}\!}X_{f} X_{f'}$ to the GLM. Here we develop an algorithm based on observations from second order HDMR expansion of *E*(*L*(*X*)|*X*), i.e., *Z*=*L*(*X*).

Following the derivation of the HDMR expansion in (10), we only need to substitute *Z*=*L*(*X*) to obtain the HDMR expansion. Now, to compute the HDMR expansion, we need the full joint distribution. However, we are almost never given the true underlying distribution parameters, and are given the training sample $\mathscr {S}$ instead. Note when working with SNP data, the number of disease associated SNPs that may affect the output may be larger than the sample size. Therefore, we may not be able to arrive at a well-defined set of distribution parameters estimates, let alone hoping the estimates to be accurate. Therefore, in the following, we present an algorithm to compute the approximate second order HDMR expansion of the log likelihood ratio directly without computing the distribution parameters for categorical dosage SNP data.

### Sobol indices, HDMR expansion, and variable selection

The Sobol indices are an extension of the *R*^2^ statistic, and can be used for global sensitivity analysis [16]. They basically explain the portion of variance explained by each set of variables. For each set of features, *u*, the total effect Sobol index and the main effect Sobol index are respectively defined as

11 $$\begin{array}{*{20}l} S(u)&=\frac{var(E(Z|X_{u}))}{var(Z)}, \end{array} $$

12 $$\begin{array}{*{20}l} S^{c}(u)&=1-\frac{var(E(Z|X_{- u}))}{var(Z)}. \end{array} $$

In other words, the total effect Sobol index describes the portion of variance that the set *u* explains, and the main effect Sobol index describes the amount of information present in *u* that is not present in any other feature.

The approximate second order HDMR expansion of Eq. (10) can also be used to analyze the extent of the effect of each feature and feature pair on the class labels. Note that if a feature is independent of the class labels then *S*(*X*_*f*_)=0 and if two features *f*_*i*_ and *f*_*j*_ do not have any interactions, i.e.,

13 $$\begin{array}{*{20}l} E\left(L(X)|X_{f_{i}f_{j}}\right)=E\left(L(X)|X_{f_{i}}\right)+E\left(L(X)|X_{f_{j}}\right), \end{array} $$

then $S\left (X_{f_{i},f_{j}}\right)=0$. The use of *S*(*X*_*f*_) for variable selection and its connection to other methodologies are discussed below. We then study how the exact HDMR expansion motivates analyzing feature pairs, and study the special case of categorical features.

#### The Sobol indices and feature filtering

In feature selection and biomarker discovery literature, univariate filters, or filters in short, refer to the family of feature selection algorithms that assess each feature individually and assign a score to each individual feature, which is then used for selecting a subset of features [17, 18]. Filters are fast, but do not take feature dependencies into account. Note in this taxonomy, univariate hypothesis tests, such as t-test, equipped with multiple testing correction are an example of filters. Other methodologies used for feature selection include multivariate filters, wrappers, and embedded methods, which are studied in more detail in [1, 17–19]. Here we study how the Sobol index for each single feature reduces to filtering, and how it connects to other filter methods.

Let *u*={*f*} be the set of single feature *f*. We have

14 $$\begin{array}{*{20}l} S(\{f\})=S_{f}=\frac{var\left(E\left(Y|X_{f}\right)\right)}{var(Y)}, \end{array} $$

15 $$\begin{array}{*{20}l} S^{c}(\{f\})=S^{c}_{f}=1-\frac{var\left(E\left(Y|X_{-f}\right)\right)}{var(Y)}. \end{array} $$

In other words, the total effect Sobol index measures how much information each feature contains about *Y*, and the main effect Sobol index measure how much information *f* carries about *Y* that is not present in any other feature. Here we prefer to use *S*_*f*_ over $S^{c}_{f}$ for three major reasons: (1) Due to the dependencies among biological features and that each individual feature might only have a very small impact on *Y*, the main effect index might be small for all features. (2) In a high-dimensional setting looking at all features but one may cause over-fitting, making it impossible to reliably measure $S^{c}_{f}$. Finally, (3) the computation cost to measure $S^{c}_{f}$ for all features can be infeasible.

Looking at *S*_*f*_, we would like to select features which affect the output the most, i.e., have large *S*_*f*_’s, which we may formulate as the following hypothesis test:

16 $$\begin{array}{*{20}l}  H_{0}: S_{f}=0 \ \ \ \ \ \ \ \ v.s. \ \ \ \ \ \ \ \ H_{1}: S_{f}>0. \end{array} $$

Note that *S*_*f*_=0 if and only if *f* is independent of *Y*. Therefore, the null of the hypothesis test of Eq. (16) can be reformulated as follows:

1. Given *f* no decision rule with less error than random decision can be built.
2. *Y* and *f* are independent.
3. *f* has the same distribution in both classes.

Note the first formulation has been used to develop methods that train a classifier/regression model, and aim to assess if it outperforms a random decision, for instance using a generalized linear model (GLM) with logit link and linear model *β*_0_+*β*_*f*_ for each *f* and outputting the *p*-value for *β*_*f*_=0. The second formulation leads to using independence criteria, such as Hilbert-Schmidt independence criterion, and the null assuming *Y* and *f* are independent. Finally, the last formulation, which may be the most popular one, leads to hypothesis tests that aim to verify if the class-conditioned distributions are different. The Kolmogorov-Simirnov (KS) test and Wilcoxon rank sum test are examples of such tests. Note that different formulations give rise to different nulls and hence different *p*-values. However, under certain assumptions one may be able to identify different tests with each other. For example, assuming (independent) Gaussian features with equal variances in both classes, linear discriminant analysis (LDA) performs better than a random decision if and only if *f* has different means in both classes, which is exactly what student-t test measures. Additionally, assuming features are independent and Gaussian with some unknown mean and variances, quadratic discriminant analysis (QDA) performs better than a random decision if *f* does not have the same mean and variances in both classes, which is exactly what the likelihood ratio test of does [20], which is studied in more detail in [21].

Fisher’s exact test and *χ*^2^-test are two popular hypothesis tests used to determine if two categorical features have similar distributions in both classes. The recently proposed optimal Bayesian filter (OBF) [22] directly measures the sample conditioned probability of a categorical variable having distributional differences across two classes, is suggested to enjoy superior performance compared with several other selection algorithms used for identifying disease associated SNPs [22]. Note we will later use OBF for feature selection in our pipeline.

#### Pairwise SNP interactions

Suppose *f*_*i*_ and *f*_*j*_ are categorical random variables, and we would like to see if a specific patten in the form of $1_{X_{f_{i}}=c_{i} \& X_{f_{j}}=c_{j}}$ carries significant information not available if we consider each feature individually. In other words, we would like to test if the second order HDMR term corresponding to feature pair *f*_*i*_,*f*_*j*_ is non-zero. Let

17 $$\begin{array}{*{20}l} k_{c_{i} c_{j}}^{f_{i},f_{j}}(y)=\frac{P\left(X_{f_{i}}=c_{i} \& X_{f_{j}}=c_{j}|y\right)} {P\left(X_{f_{i}}=c_{i}|y\right)P\left(X_{f_{j}}=c_{j}|y\right)}. \end{array} $$

Note that

18 $$\begin{array}{*{20}l} cov\left(1_{X_{f_{i}}=c_{i}},1_{X_{f_{j}}=c_{j}}|y\right) &= E\left(1_{X_{f_{i}}=c_{i}}\times1_{X_{f_{j}}=c_{j}}|y\right)\\&-E\left(1_{X_{f_{i}}=c_{i}}|y\right)E\left(1_{X_{f_{j}}=c_{j}}|y\right)  \\ &= \left(k_{c_{i} c_{j}}^{f_{i},f_{j}}(y)-1\right)P\left(X_{f_{i}}=c_{i}|y\right)\\&\quad P\left(X_{f_{j}}=c_{j}|y\right). \end{array} $$

Therefore, we would like to see if $k_{0}^{f_{i},f_{j}} \neq k_{1}^{f_{i},f_{j}}$. Thereby, by a pairwise SNP interaction we understand the non-zero second order HDMR terms that involve two distinct SNPs, i.e., are not present in the first order expansion. Here we see that such terms correspond to SNP pairs with unequal correlations between the two classes. Note that a linear additive model looking only at individual SNPs is not sufficient to compute the log likelihood ratio for such correlated categorical features. This is also in line with previous definitions proposed for quantifying SNP interactions, e.g., [6, 23].

Now to compute the *p*-value associated with each fixed SNP pair pattern we use Fisher’s r to z transformation and approximate the distribution of the null $\left (k_{0}^{f_{i},f_{j}} = k_{1}^{f_{i},f_{j}}\right)$ by the standard normal distribution. We compute the statistics

19 $$\begin{array}{*{20}l} z^{f_{i},f_{j}}_{y}=0.5 \log\left(\frac{1+\hat{\rho}^{f_{i},f_{j}}_{y}}{1-\hat{\rho}^{f_{i},f_{j}}_{y}} \right) \end{array} $$

for *y*=0,1, where $\hat {\rho }^{f_{i},f_{j}}_{y}$ is the estimate of correlation coefficient from data. We then compute

20 $$\begin{array}{*{20}l} Z^{f_{i},f_{j}}=\frac{z^{f_{i},f_{j}}_{1}-z^{f_{i},f_{j}}_{0}}{\sqrt{\frac{1}{n_{0}-3}+\frac{1}{n_{1}-3}}}, \end{array} $$

where *n*_*y*_ is sample size in class *y*. $Z^{f_{i},f_{j}}\phantom {\dot {i}\!}$ approximately follows the standard normal distribution, which we use to compute *p*-values. We hereafter call this method *fixed pattern test* (FPT).

## Algorithm

Here we describe the algorithm developed for classifier design.

### Initial filtering

Here we outline the pre-processing we do on the categorical SNP data. When working with SNP data, many times dosage values, i.e., the number of minor alleles, are reported which take values in {0,1,2}. Note that many SNPs can be dominant or recessive. A dominant SNP is one for which a SNP with one minor allele behaves similar to a SNP with two minor alleles, and a recessive SNP is one for which a SNP with one minor allele behaves similar to a SNP with non minor alleles. Therefore, we do a first phase pre-processing, and transform each SNP to two “binarized SNP”s. For each SNP, we create two auxiliary features, one the indicator of the presence of a minor allele, and another the indicator of two minor alleles. This way, if only the first auxiliary feature is used then we are dealing with a dominant SNP, if only the second auxiliary feature is used we have a recessive SNP, and if both are used the SNP does not fall into only one of the two categories, for instance we may have an additive SNP. Examples of different SNP models and a discussion on them can be found in [24, 25].

By this preprocessing, if *k* SNPs are observed, we have |*F*|=2*k* binarized SNPs. We hereafter assume all SNPs are already binarized, i.e., for each feature *f*∈*F* we have *C*_*f*_={0,1}, instead of using the family of constraints *Q*^*f*^. Note that we only do so for notational convenience.

As mentioned above, given that many studies may measure several hundred thousand or a few million SNPs, they all cannot be directly inputted to a classifier, particularly that we aim to account for pairwise SNP interactions, i.e., the second order terms of the HDMR expansion. A first phase filtration is typically inevitable to reduce computational complexity of classifier design, i.e., the training of the classification algorithm. Here we use the recently proposed optimal Bayesian filter (OBF) [22], to rank binarized SNPs, and we pick the top *D* features for classifier design. Note *D* is a parameter that can be determined later through cross validation, or be chosen as a large value so that most disease associated SNPs are captured. Note we have chosen OBF as it has outperformed many of the currently used methods for selecting disease SNPs, including the popular *χ*^2^-test [22].

### Classification algorithm

Here we design the classifier used to label observations. Note here we are using binarized SNPs as our features. In the training, given the labeled training data and selected binarized SNPs of the preprocessing step, the algorithm assigns coefficients to all SNPs and SNP pairs. Note *D* might be large, and given *D* features, there are 0.5*D*(*D*−1) SNP pairs to use. Furthermore, there may be weak individual SNPs or SNP pairs that are so weak that not including them in the final classifier might actually improve performance. Therefore, in our classifier design we remove features and feature pairs that are too weak. The classifier design process can be broken to the following steps: (1) feature pair construction, (2) removing weak features, (3) removing weak feature pairs, (3) merging feature pairs into blocks, and (4) estimating classifier parameters and obtaining the risk function.

#### Feature pair construction

**Fig. 1:**
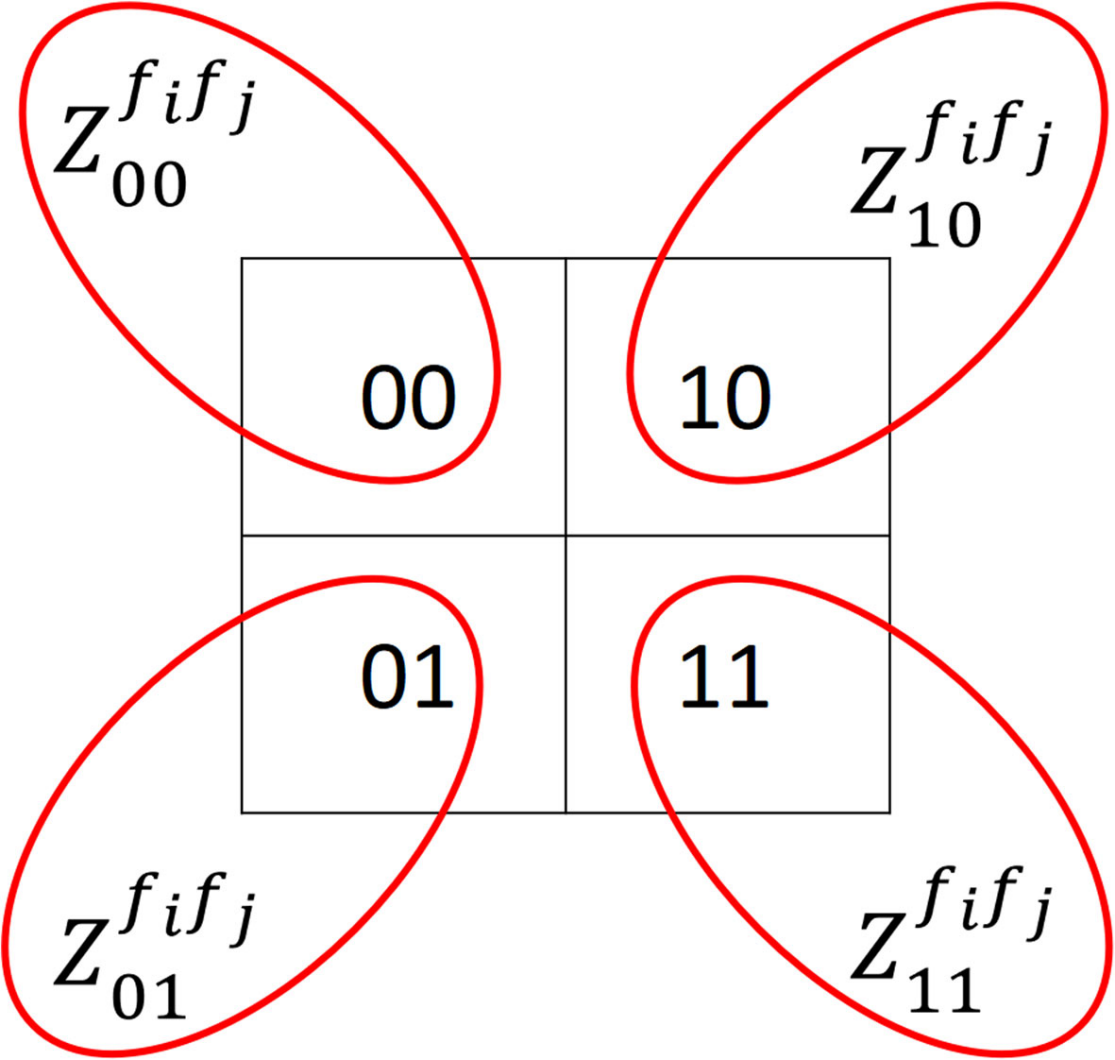
An illustration of creating four binarized feature pairs $Z^{f_{i},f_{j}}_{c_{1},c_{2}}$ given binarized features *f*_*i*_ and *f*_*j*_. Each square denotes a pairwise value of binary SNPs *f*_*i*_ and *f*_*j*_, and how they are denoted as feature pairs $Z^{f_{i},f_{j}}_{c_{i},c_{j}}$

Given that we have binarized SNP values, for two features *f*_*i*_ and *f*_*j*_, we have four feature pairs of the form $Z^{f_{i},f_{j}}_{c_{i},c_{j}}=1_{\left \{X_{i}=c_{f_{i}} \& X_{j}=c_{f_{j}}\right \}}$. Each of the created $Z^{f_{i},f_{j}}_{c_{i},c_{j}}$’s is hereafter called a *feature pair*. Figure 1 illustrates how to generate feature pairs $Z^{f_{i},f_{j}}_{c_{i},c_{j}}$ from binarized SNPs.

#### Removing weak features

**Fig. 2:**
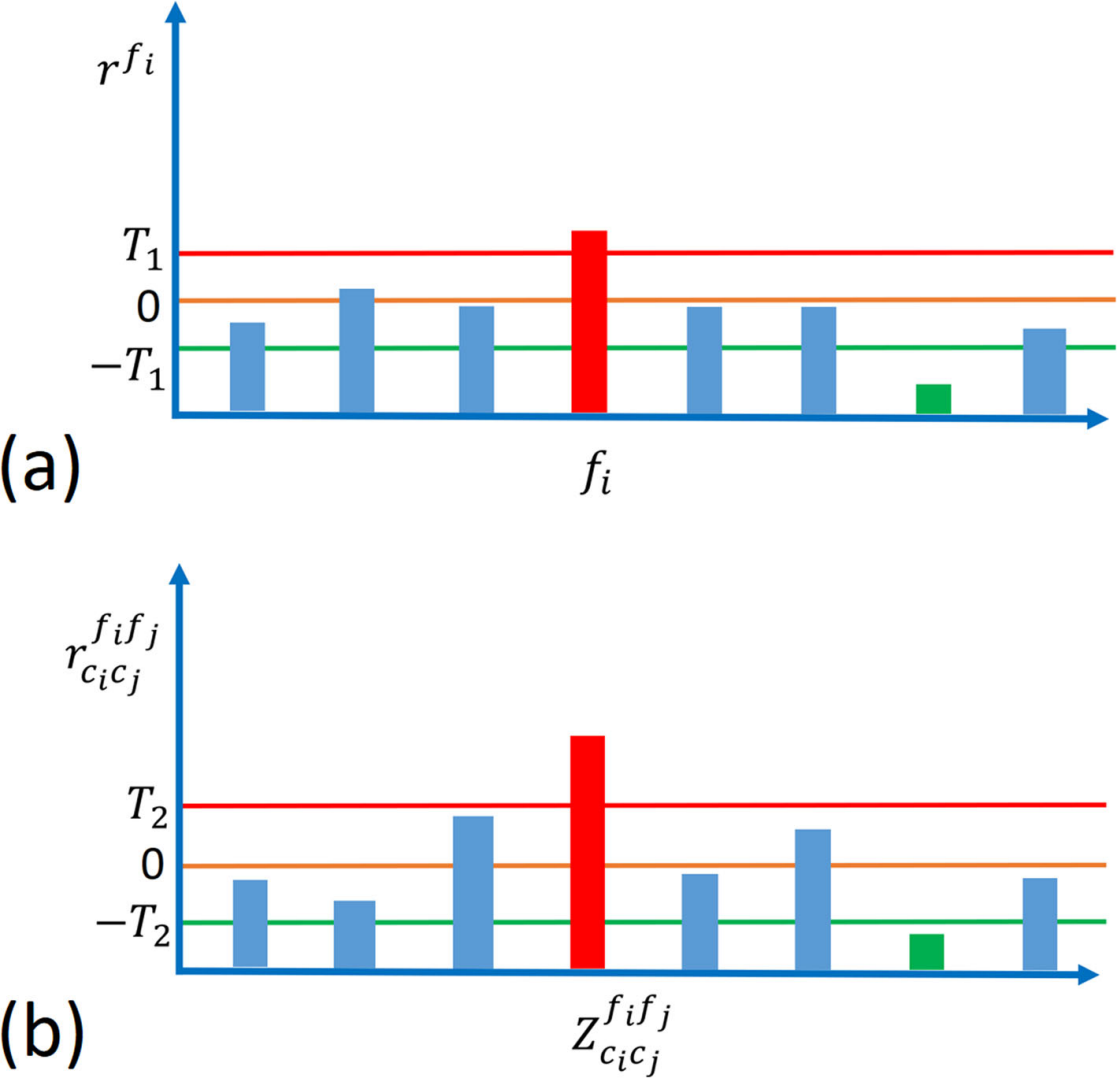
Illustration of removing weak (a) features and (b) feature pairs. **a** Features for which |*r*^*f*^|<*T*_1_ are removed. Features for which *r*^*f*^>*T*_1_ are risk increasing features, and features for which *r*^*f*^<−*T*_1_ are the risk decreasing features. **b** Similarly, features pairs for which $\left |r^{f_{i}f_{j}}_{c_{i}c_{j}}\right |<T_{2}$ are removed. Feature pairs for which $r^{f_{i}f_{j}}_{c_{i}c_{j}}>T_{2}$ are risk increasing feature pairs, and feature pairs for which $r^{f_{i}f_{j}}_{c_{i}c_{j}}<-T_{2}$ are risk decreasing. Red and green, respectively, denote risk increasing and risk decreasing features and feature pairs

Here we remove features that are too weak to be used in the classifier. For each feature, *f*, we find the risk associated to it, *r*^*f*^. We first compute $v^{f}=\max \left \{|\log \hat {p}^{f}_{1,1}/\hat {p}^{f}_{1,0}|, | \log \hat {p}^{f}_{0,1}/\hat {p}^{f}_{0,0}| \right \}$, where $\hat {p}^{f}_{c,y}$ is the posterior probability of *X*_*f*_=*c* in class *y* and is obtained through OBF. If $\hat {p}^{f}_{c,1}>\hat {p}^{f}_{c,0}$ for the pattern *c* obtaining the maximum in the definition of *v*^*f*^ we set *r*^*f*^=*v*^*f*^; otherwise, *r*^*f*^=−*v*^*f*^. We assign the zero coefficient, i.e., remove, features for which |*r*^*f*^|<*T*_1_. Note *T*_1_ a model parameter. Note features for which $\hat {p}^{f}_{1,0}>\hat {p}^{f}_{1,0}$ are called risk increasing or positive risk features, and features for which $\hat {p}^{f}_{1,0}<\hat {p}^{f}_{1,0}$ are called risk decreasing features. Figure 2a illustrates this process for single features.

#### Removing weak feature pairs

We then filter out feature pairs that are too weak. We again follow a procedure similar to our process for removing single features. For each feature pair *f*_*i*_ and *f*_*j*_ define $r^{f_{i}f_{j}}_{c_{i}c_{j}}=\log \left (\hat {p}^{f_{i}f_{j}}_{c_{i}c_{j}}(1)/\hat {p}^{f_{i}f_{j}}_{c_{i}c_{j}}(0)\right)-\log \left (\hat {p}^{f_{i}}_{c_{i},1}/\hat {p}^{f_{i}}_{c_{i},0}\right)-\log \left (\hat {p}^{f_{j}}_{c_{j},1}/\hat {p}^{f_{j}}_{c_{j},0}\right)$ where $\hat {p}^{f_{i}f_{j}}_{c_{i}c_{j}}(y)$ is the sample conditioned probability that feature pair *f*_*i*_,*f*_*j*_ satisfies $X_{f_{i}}=c_{i}$ and $X_{f_{j}}=c_{j}$ in class *y*. Again, feature pairs for which $\left |r^{f_{i}f_{j}}_{c_{i}c_{j}}\right |<T_{2}$ are removed, i.e., are assigned zero weight in the classification rule. Note again feature pairs for which $\hat {p}^{f_{i}f_{j}}_{c_{i}c_{j}}(1)>\hat {p}^{f_{i}f_{j}}_{c_{i}c_{j}}(0)$ are called risk increasing or positive risk feature pairs, and feature pairs for which $\hat {p}^{f_{i}f_{j}}_{c_{i}c_{j}}(1)<\hat {p}^{f_{i}f_{j}}_{c_{i}c_{j}}(0)$ are called risk decreasing or negative risk feature pairs. Here we have defined $r^{f_{i}f_{j}}_{c_{i}c_{j}}$ so that the interaction of a feature pair comprised of two independent features would not enter the classification rule. Figure 2b illustrates this process for feature pairs.

#### Feature block construction

Recall given *D* features, there are 0.5*D*(*D*−1) feature pairs. Although our filtering removes many feature pairs, there still could be too many feature pairs to be easily used for classifier design. In addition, since (a) SNPs may be heavily correlated, (b) a feature may occur in many feature pairs, and (c) the binarization scheme described in the pre-processing step creates two binary features for each SNP, the binary features might be heavily correlated, creating dependencies among feature pairs. In other words, given that a specific pattern for a feature pair is observed, one may be able to estimate the value of many other feature pairs. For example, given we observe $1_{\left \{X_{f_{i}}=1\right \}}=0$ for a specific feature pair that uses $1_{\left \{X_{f_{i}}=2\right \}}$, we can easily conclude all feature pairs that assume $1_{\left \{X_{f_{i}}=2\right \}}$ are zero. These dependencies exacerbate classifier design. Therefore, we propose the following procedure to reduce the number of weights to estimate. Note we could have used any other community detection algorithm instead; however, we observed the following procedure works well, and enjoys low computation cost.

**Fig. 3:**
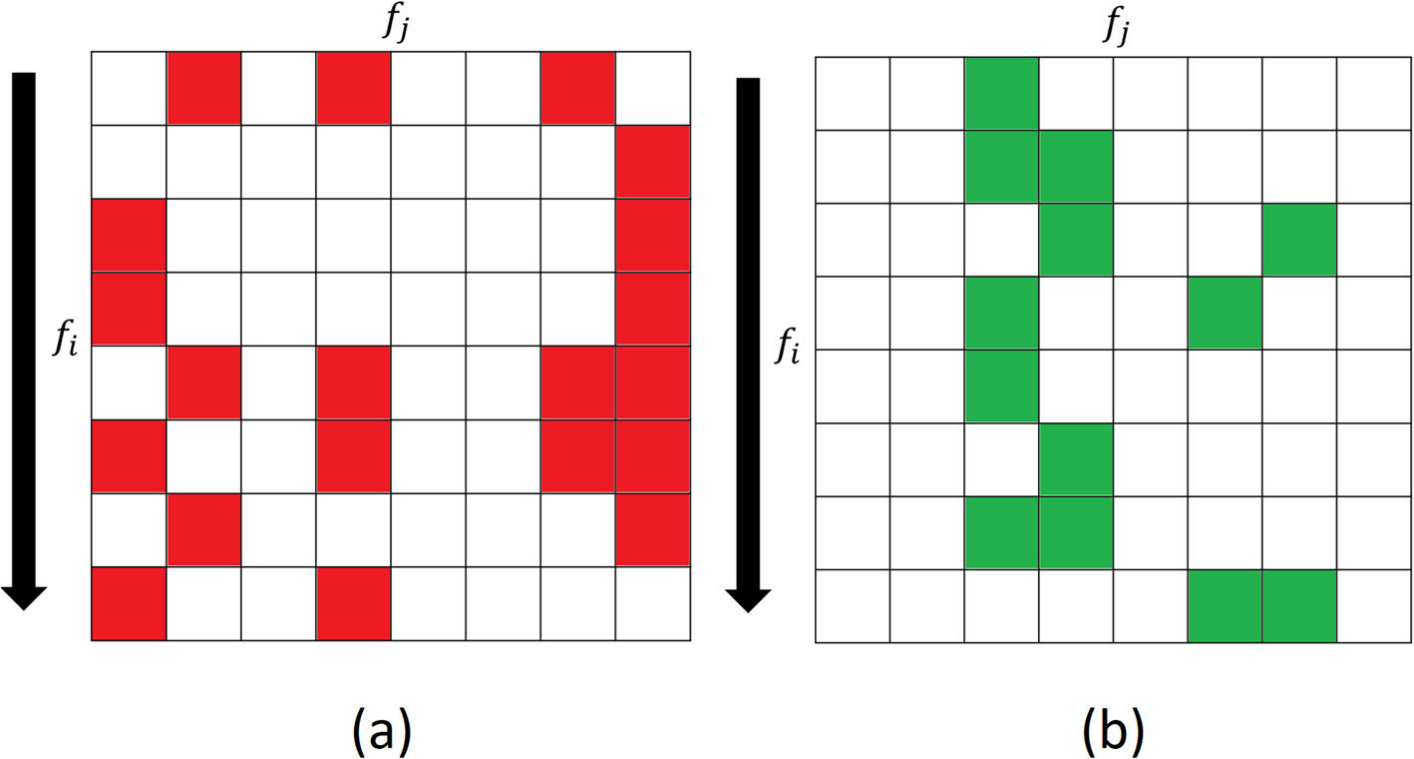
Illustration of constructing **a** risk increasing and **b** risk decreasing blocks. Each red/green square in row *f*_*i*_ and column *f*_*j*_ is selected as a risk increasing/decreasing feature pair $\left (\left |r^{f_{i}f_{j}}_{c_{i}c_{j}}\right |>T_{2}\right)$ to construct risk increasing/decreasing block $P^{f_{i}}_{c_{1},c_{2}}$/$N^{f_{i}}_{c_{1},c_{2}}$. Finally white squares correspond to feature pairs that are removed, i.e., have risks less than *T*_2_

We consider blocks of feature pairs of the following forms for each feature *f*_*i*_ and pattern *c*_*i*_*c*_*j*_:

$$\begin{array}{*{20}l} P^{f_{i}}_{c_{1}c_{2}}&=\left\{Z^{f_{i}f_{j}}_{c_{1}c_{2}} : j \neq i, r^{f_{i}f_{j}}_{c_{1}c_{2}}>T_{2} \right\},  \\ N^{f_{i}}_{c_{1}c_{2}}&=\left\{Z^{f_{i}f_{j}}_{c_{1}c_{2}} : j \neq i, r^{f_{i}f_{j}}_{c_{1}c_{2}}<-T_{2} \right\}, \end{array} $$

where *P*’s and *N*’s are collections of risk increasing and risk decreasing feature pairs, respectively. Figure 3 depicts this process. Afterwards, given an observation *X*, we report the ratio of feature pairs in each block which take value one. Among the constructed blocks, we again remove “weak blocks”, i.e., for each block *A*, irrespective of being risk increasing or risk decreasing, we compute *r*^*A*^, the logarithm of the expected ratio of observed patterns of block *A* in class 1 versus class 0. We then remove blocks for which |*r*^*A*^|<*T*_3_. Again, *T*_3_ is threshold that will be chosen through cross validation.

We observed this approach to (a) reduce the number of parameters to estimate when *D* is large, and (b) improve prediction performance. Note that this approach is equivalent to decomposing $w_{f_{i}f_{j}}^{c_{f_{i}} c_{f_{j}}}$ to two terms, one for the block $A^{f_{i}}_{c_{i} c_{j}}$ and another for block $A^{f_{j}}_{c_{j} c_{i}}$, where *A* is either *P* or *N*, and assuming all features pairs in $A^{f_{i}}_{c_{i} c_{j}}$ have the same decomposed coefficient in their expansion. Here, in the simulations, we observed such assumptions improves classification performance when features, i.e., SNPs, are correlated, but leave a mathematical analysis of such assumption on the classifier performance for future work.

Now, given observation *X*, we construct the vector *V*(*X*), comprised of each feature value, and the ratio of observed patterns in risk increasing and risk decreasing blocks. The vector *V*(*X*) shall be used in the next section to assign a “risk” to observation *X*.

#### Estimating classifier parameters

To complete our classifier construction, we need to estimate HDMR coefficients. Note given our construction of blocks in the previous section, we now only need to find a vector *b* so that we may write that *E*_2_(*L*(*X*)|*X*)≈*E*(*L*(*X*))+*b*.*V*(*X*). However, we may be dealing with an ill-posed problem due the number of coefficients to estimate being larger than the sample size. Note that although the HDMR expansion of the log likelihood ratio is unique, we mostly compare it with a threshold to assign a label to a newly observed point. Therefore, in many cases it is acceptable to work with a affine transformation of the log likelihood ratio. In other words, although not being able to find the exact second order HDMR expansion of the log likelihood ratio is not desirable, it is not catastrophic either, as any affine transformation of the log likelihood ratio can be used as an equally good decision rule.

**Fig. 4:**
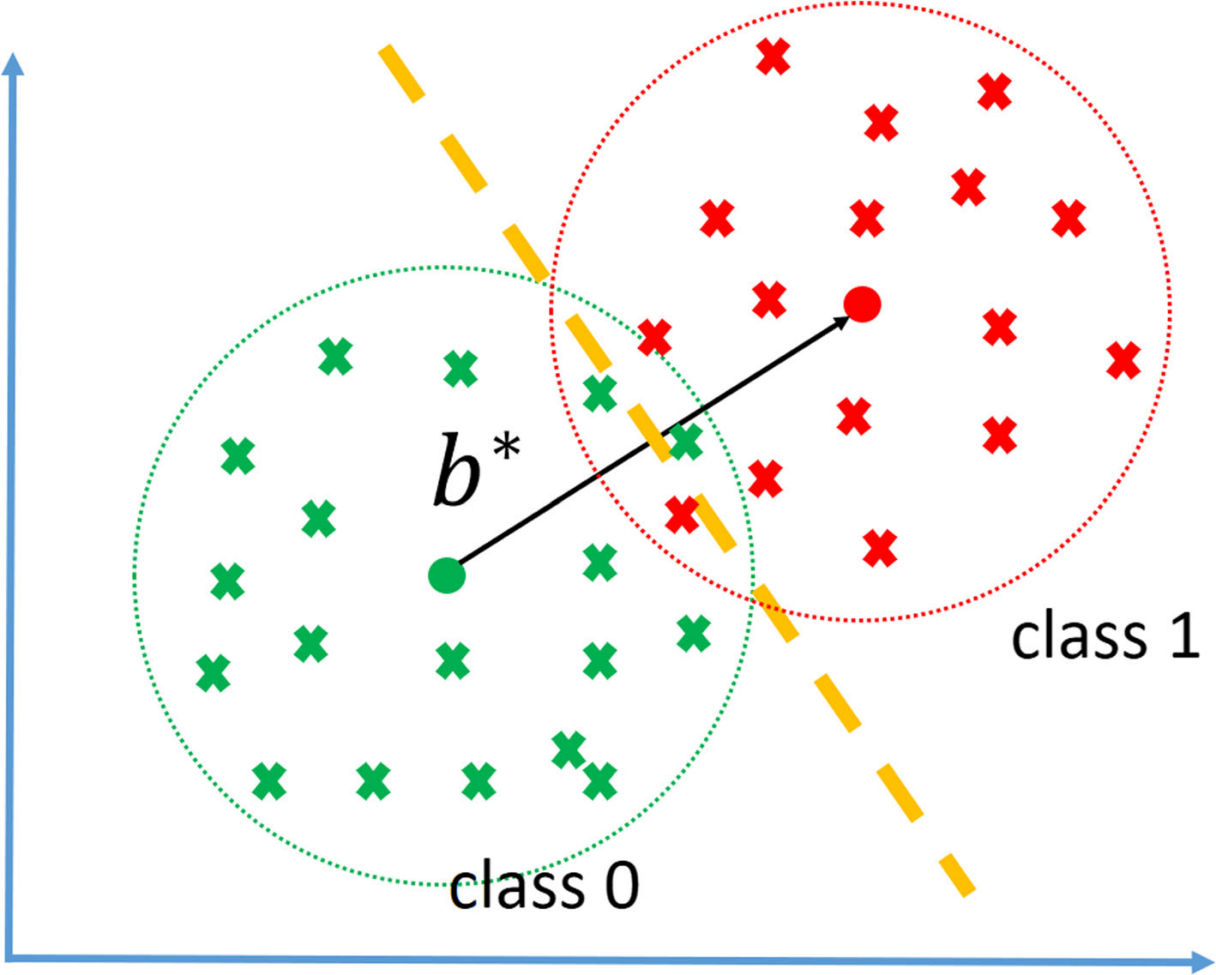
The illustration of the *b*^∗^ selection process. Given *V*(*X*) vectors for the two classes, denoted by red and green crosses, the *b*^∗^ is chosen to maximize the distance between the center of projections of *V*(*X*) vectors on *b*

To circumvent the ill-posed problem, we use an objective function which is a variation of objective functions mostly studied in the compressed sensing literature [26] that aim to estimate a sparse signal given 1-bit quantized observations. In other words, optimization problems and formulations that aim to estimate vector *a* from *n* observations of the form {(*x*_*i*_,*y*_*i*_):*i*=1:*n*} where *y*=*sign*(*a*·*x*) and “ ·” denotes inner product. Connections between these objectives and a convex relaxation to the logistic regression problem is discussed in [27]. Extensions that additionally consider noisy measurements in the form of random flips, i.e.,

$$\begin{array}{*{20}l} y=\left\{\begin{array}{lc} sign(a\cdot x)\ \ \ \ \ \ \text{with probability} \ 1-\epsilon,  \\ -sign(a\cdot x)\ \ \ \ \text{with probability} \epsilon, \end{array}\right. \end{array} $$

are studied in [27]. Finally, [28] studies extensions to non-Gaussian features. Note in [27] it is shown that up to a constant the error in recovering the signal *a* matches the minimax error for the unquantized compressed sensing problem. We use the following optimization problem to solve for the weights we wish to use.

21 $$\begin{array}{*{20}l}  b^{*}=\underset{b}{argmax} \frac{1}{n_{1}} \sum\limits_{X \in \mathscr{S}_{1}} b\cdot V(X) - \frac{1}{n_{0}} \sum\limits_{X \in \mathscr{S}_{0}} b \cdot V(X), \end{array} $$

where $\mathscr {S}_{y}$ is the portion of data in class *y*. Figure 4 depicts how *b*^∗^ is selected given vectors *V*(*X*) for the training data. Heuristically speaking, given a feature vector in the form of log likelihood ratios of partial observations *x*_*u*_, here we find weights that maximize the distance between the average points of each class. The heuristic for using such objective is as follows: the HDMR expansion obtains the weights that result in the “best” low dimensional representation, i.e., we find the mean square error (MSE) estimate of the log likelihood ratio. The underlying reason we do so is that we believe the HDMR expansion of the log likelihood ratio gives a us a model that enjoys a low prediction error. On the other hand, weights that maximize the distance between the projections of the center points of the two classes to a one dimensional space should also yield low prediction error. Hence, such objective should result in a model that is close to the HDMR expansion. Note that in special cases, for instance independent Gaussian features with equal variances in both classes, we can actually prove that such approach minimizes the prediction error.

Given *b*^∗^ we have everything need for classification. Given a new observation *X* we find *R*(*X*)=*b*^∗^·*V*(*X*), and we assign class label $\hat {Y}=1_{R(X)>T}$, for threshold *T*. Note the thresholds *T*_1_, *T*_2_, and *T* are parameters of the model, and will be selected through the validation process, for instance, by cross validation. We hereafter call the resulting classifier built for categorical *X* as *linear approximation for block second order HDMR expansion of categorical observations* (LABS-HDMR-CO). The pseudo-code of LAS-HDMR-CO is provided in Algorithm 1.


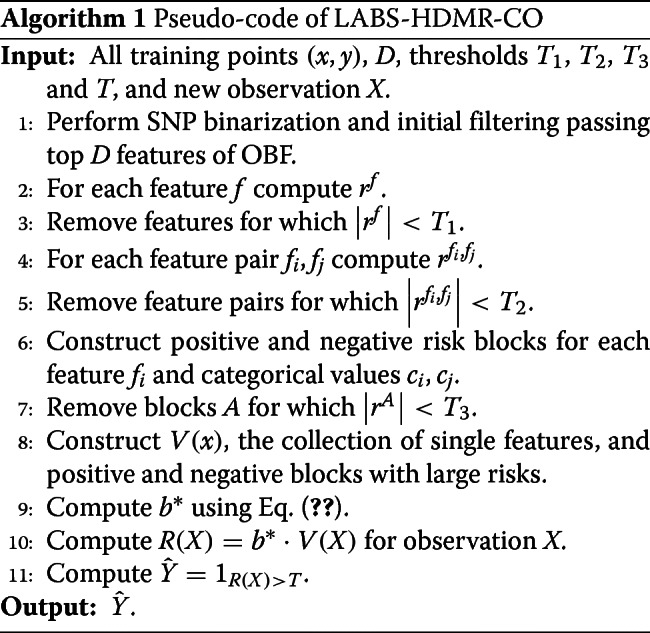


## Results

Here we use a model developed to generate SNP data to evaluate the performance of LABS-HDMR-CO, and compare it with several popular methods used for binary classification. We consider three datasets, a dataset based on the HAPGEN2 project, a lung cancer dataset, and a breast cancer dataset. OBF takes *π*(*f*), the prior probability a SNP is disease associated, and hyperparameter *α* describing the Dirichlet prior on each categorical SNP value as input. We assume *π*(*f*) is constant for all features, hence not affecting the ranking, and set *α*=[2, 2] for each binarized SNP used with LABS-HDMR-CO and *α*=[1, 1, 1] for each non-binarized SNP used with other classification rules. Note the choice of *α* to be the all one vector simplifies to a uniform prior. As LABS-HDMR-CO uses feature pairs, a uniform prior on binarized SNP patterns suggests *α*=[2, 2].

### HAPGEN2 data

Here we generate data from the HAPGEN2 project [9], reporting SNP values for more than 3.9 million SNPs, which we then convert to dosage. This dataset is generated by fixing one or two SNPs on each chromosome to be disease associated. The generated dataset contains 2000 controls (class 0), and 1000 cases (class 1). We randomly select 900 points in each class for training, and the rest is used as test data. We iterate 100 times, and measure the area under curve (AUC) of the receiver operator characteristic (ROC) as our performance metric

In addition to LABS-HDMR-CO, we use the non-processed data, use OBF for feature selection to select top features, and use several variants of GLMs with probit link for further selection and classification. We use a probit model that uses top 1000 features with LASSO (*L*_1_) penalty *λ* and another that uses top 500 features with elastic net putting equal weights on *L*_1_ and *L*_2_ penalties with penalty coefficient *λ*. We also use a variant that accounts for pairwise SNP interactions by considering terms of the form *X*_*i*_*X*_*j*_ using top 50 features and *L*_1_ penalty *λ*. We only use the top 50 features (see Tables 1, 2) for the variant accounting for pairwise SNP interactions so that the total number of regressors to use in the regression model is comparable to the linear variants. We observed that larger values of features in the probit link accounting for pairwise interactions drastically increases runtime, causing infeasible computation cost. Finally, we also implement a naive Bayes classifier using top 1000 features.

**Table 1:** Several top SNPs and their associated risk for the HAPGEN2 project

Rank	SNP id	Chromosome	Location	Risk	+/− Risk	Recessive
1	rs1982151	9	85807085	5.38	-	TRUE
2	rs796004	9	85784618	5.38	+	FALSE
3	rs296887	9	85784890	5.38	+	FALSE
4	rs296889	9	85785391	5.38	+	FALSE
5	rs296890	9	85785551	5.38	+	FALSE
6	rs796003	9	85785621	5.38	+	FALSE
7	rs296893	9	85788010	5.38	+	FALSE
8	rs11140325	9	85824855	5.38	-	TRUE
9	rs10868080	9	85816589	5.38	-	TRUE
10	rs296888	9	85785318	5.34	+	TRUE
40	rs861539	14	103235506	4.4	-	FALSE
41	rs861534	14	103238454	4.4	-	TRUE
42	rs861531	14	103242560	4.4	-	FALSE
43	rs8018979	14	103178840	4.39	-	FALSE
44	rs11849259	14	103186074	4.39	-	FALSE
45	rs3783404	14	103188251	4.39	-	FALSE
46	rs55885592	14	103195777	4.39	-	FALSE
47	rs56660916	14	103195890	4.39	-	FALSE
48	rs57218990	14	103197760	4.39	-	FALSE
49	rs2403205	14	103213138	4.39	-	FALSE
50	rs709400	14	103219228	4.39	-	FALSE

**Table 2:** Top 25 SNP pairs and their associated risk

Rank	SNP ID	Recessive	Value	SNP ID	Recessive	Value	+/− Risk	Risk
1	9-85652897	FALSE	1	9-85439030	FALSE	0	+	1.43
2	9-85652915	FALSE	1	9-85439030	FALSE	0	+	1.43
3	9-85652897	FALSE	1	9-85524813	FALSE	0	+	1.4
4	rs12897511	TRUE	0	9-85652897	FALSE	1	+	1.35
5	9-85779070	FALSE	1	rs12346234	FALSE	0	+	1.34
6	9-85652897	FALSE	1	rs861539	TRUE	0	+	1.33
7	9-85652897	FALSE	1	rs861534	TRUE	0	+	1.33
8	9-85652897	FALSE	1	rs861531	TRUE	0	+	1.33
9	9-85652897	FALSE	1	rs10135248	TRUE	0	+	1.33
10	9-85652897	FALSE	1	rs3915733	TRUE	0	+	1.33
11	9-85652897	FALSE	1	rs8005885	TRUE	0	+	1.33
12	9-85652897	FALSE	1	rs8018979	TRUE	0	+	1.33
13	9-85652897	FALSE	1	rs11849259	TRUE	0	+	1.33
14	9-85652897	FALSE	1	rs3783404	TRUE	0	+	1.33
15	9-85652897	FALSE	1	rs55885592	TRUE	0	+	1.33
16	9-85652897	FALSE	1	rs56660916	TRUE	0	+	1.33
17	9-85652897	FALSE	1	rs57218990	TRUE	0	+	1.33
18	9-85652897	FALSE	1	rs2403205	TRUE	0	+	1.33
19	9-85652897	FALSE	1	rs709400	TRUE	0	+	1.33
20	9-85652897	FALSE	1	rs861548	TRUE	0	+	1.33
21	9-85652897	FALSE	1	rs11624505	TRUE	0	+	1.33
22	9-85652897	FALSE	1	rs61995780	TRUE	0	+	1.33
23	9-85652897	FALSE	1	rs861536	TRUE	0	+	1.33
24	rs861539	TRUE	0	9-85828929	TRUE	0	+	1.32
25	rs861539	TRUE	0	rs7039458	TRUE	0	+	1.32

Indeed, it is an advantage of LABS-HDMR-CO that can incorporate several hundred features in its regression model while accounting for pairwise SNP interactions with reasonable computational burden. Finally, our reasons to choose the probit link over the more popular logit link are 3 fold: (1) In the data generation model the risk of an SNP mutation is modeled as additional linear risk; corresponding to the logit link. Therefore, models based on the logit link get an unfair advantage that they exactly match the data generation model; while in reality almost always the assumed model deviates from reality. (2) We observed the computational cost to train a probit link is much less, about a third, of the logit link. Hence, to reduce computational cost of the GLM variants we compare with, we selected the probit link. (3) In practice the probit link behaves similar to the logit link, which is not surprising as the sigmoid and the cumulative distribution function (CDF) of the standard normal distribution are rather similar. Hence this choice better illustrates how slights deviations in the assumed link might affect performance of a GLM when dealing with SNP data.

Finally, note that all GLM variants and naive Bayes use OBF assuming three categories, i.e., three dosage values, for ranking features. We tested the popular *χ*^2^-test as well, and obtained lower AUCs for the GLMs and naive Bayes. This results strengthens the observations made in [22] that OBF provides better feature rankings compared several other methods, including the popular *χ*^2^-test.

**Fig. 5:**
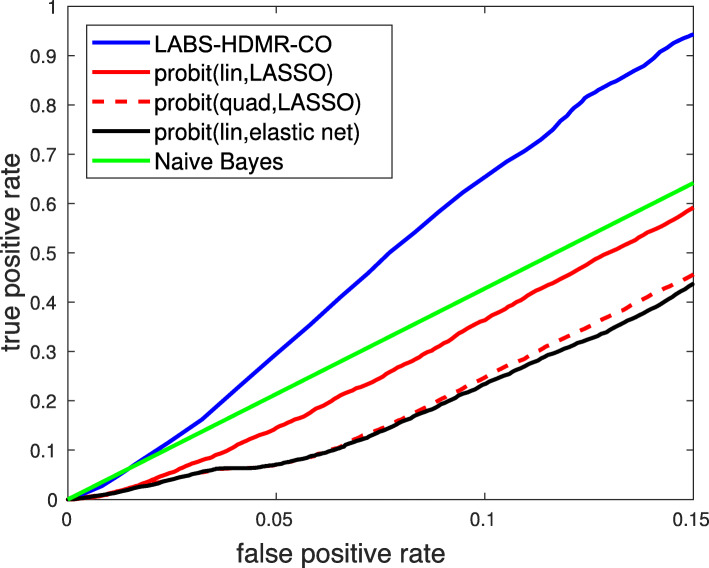
ROC curve of different classification rules for the generated data based on HAPGEN2 project [9]

The AUCs for LABS-HDMR-CO, the linear probit model using *L*_1_ penalty (probit(lin,LASSO)), second order probit model with *L*_1_ penalty (probit(quad,LASSO)), linear probit using elastic net (probit(lin,elastic net)) and naive Bayes are 91.03*%*, 84.52*%*, 86.44*%*,84.28*%*, and 86.83*%*, respectively. The larger AUC of LABS-HDMR-CO suggests it enjoys superior overall performance, i.e., LABS-HDMR-CO should typically enjoy a higher probability of detection for a fixed false alarm rate value. Figure 5 plots the ROC curve of the classification algorithms. For the GLMs we tested *λ*=0.01:0.01:0.2, and for each variant report the AUC of the *λ* with superior performance, i.e., highest AUC. Also, the parameters of LABS-HDMR-CO are chosen through cross validation. As the results suggest LABS-HDMR-CO enjoys superior performance compared with other algorithms. In particular, for false positive rates larger than 2% LABS-HDMR-CO enjoys higher probability of detection compared with all other algorithms. For small false alarm rates though, naive Bayes had the highest true positive rate, but was closely followed by LABS-HDMR-CO. While the ROC curve of probit(lin,LASSO) seems to monotonically increase with respect to false positive rate, true positive rate of probit(quad,LASSO) and probit(lin,elastic net) seem to only infinitesimally increase for false positive rates between 0.04 and 0.05. Given that ROC curves must be concave, we initially believed this might be an artifact of not enough iterations (here being 100); however, simulations only implementing these two GLMs with more iterations resulted in rather similar graphs. However, in many iterations (using MATLAB’s default settings) the warning of reaching maximum number of iterations was reported for these two methods. Therefore, this may be an artifact of the limited number of iterations or numerical instabilities of the training stage.

Finally, we observed the runtime of LABS-HDMR-CO using 1000 SNPs is comparable to the probit variant using 500 SNPs and elastic net. This suggests LABS-HDMR-CO is extremely fast for a method that accounts for pairwise SNP interactions. Note given 1000 SNPs there are about 500,000 SNP pairs to evaluate. However, LABS-HDMR-CO has more parameters to tune via cross validation, its total runtime is more than a GLM with one tunable parameter. Note in this work we did not test an elastic net probit that also optimizes over *α*, the relative weights between *L*_1_ and *L*_2_ penalties; however, we expect the two dimensional search for such model might result in computation costs comparable to LABS-HDMR-CO.

Finally, we use all of data, to find the top SNPs and SNP pairs with largest risks, i.e., *r*^*f*^ and $r^{f_{i}f_{j}}_{c_{i}c_{j}}$, respectively. Using Fisher’s exact test and bounding the false discovery rate (FDR) by 5% using the Banjamini-Hochberg procedure [29] 785 SNPs are significant. Also, using FPT for identifying significant pairwise SNP interaction patterns, among the $4\times \binom {1000}{2} \approx 2 \times 10^{6}$ patterns to check, 1046864, about 52.4*%* of all tests, are significant when bounding FDR by 5%. Given than many selected SNPs are on the same chromosome this is not surprising. Furthermore, we observed that all single SNPs that were not significant after bounding FDR are present in at least one SNP pair, emphasizing the importance of considering pairwise SNP interactions See for details.

### Lung cancer

**Fig. 6:**
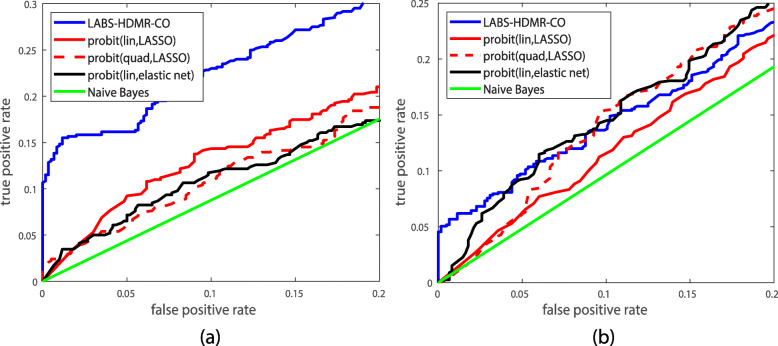
ROC curve of different classification rules for the **a** lung cancer and **b** breast cancer datasets

Data obtained in [30] is deposited on gene expression omnibus (GEO) [31] with accession number GSE33355. It contains 61 sample pairs of cancer and normal lung tissue specimen from non-smoking females collected at national Taiwan university hospital and Taichung veterans general hospital. The data is based on the GPL6801 platform and measures dosage values of 909622 SNPs. In our evaluation healthy and cancerous tissue specimen comprise classes 0 and 1, respectively. Thereby, the goal is to determine if a test point is normal or cancerous. Given the dataset we randomly select 55 points in each class for training, use the rest for testing, and iterate 100 times. Figure 6a plots the ROC curve of different methods. We observe that LABS-HDMR-CO enjoys a higher true positive rate for each given false positive rate, suggesting its superior performance on this dataset. Cross validation sets *D*=900, *T*_1_=0.25, *T*_2_=1.2, and *T*_3_=0.8. Linear and quadratic probit with LASSO penalty set *λ* to 0.2 and 0.02, respectively, and the linear probit with elastic net sets *λ*=0.13.

Tables 3 and 4 list the top SNPs and SNP pairs with largest risks used by LABS-HDMR-CO. Although cross validation suggests using *D*=900 SNPs for prediction, the Fisher’s exact test using the Benjamini-Hochberg [29] procedure for FDR correction suggests that only looking at the SNPs used for prediction, only the top 250 are significant bounding FDR by 5%. Although the remaining SNPs are not significant, their net effect is an improvement in prediction which may be due to agglomerating them as individual SNPs, or specific SNP pair intereactions among them that should be considered together as a marker family to observe their effect. Going back to the literature we observe many of the top SNPs and SNP pairs, or the genes they belong to, are shown or suggested to be affected in lung cancer. For example, the top SNP rs9493858 is located on the SGK1 gene, which is suggested to be affected in lung cancer in several studies [32, 33]. Looking at the top SNP pairs, we observe the second highest SNP pair map to NXN and MEOX2 genes, suggesting their interaction might be key to understanding lung cancer. Interestingly, mutations in NXN has been associated with colon cancer in east Asian populations [34], but its role in lung cancer requires further investigation. Furthermore, MEOX2 is also suggested to be affected in lung cancer [35]. In this dataset, except for one SNP pair, all risks are positive. In other words, certain mutations increase the cancer risk; however, the data does not suggests any candidate mutations that seem to further help healthy people guard against lung cancer.

**Table 3:** Several top SNPs and their associated risk for the lung cancer dataset

Rank	SNP ID	Chromosome	Location	Risk	+/− Risk	Recessive	Gene
1	rs9493858	6	134535159	1.9	+	FALSE	SGK1
2	rs11768533	7	27560904	1.56	+	FALSE	-
3	rs3134492	8	96699596	1.54	-	FALSE	C8orf37-AS1
4	rs9893755	17	79187432	1.47	+	FALSE	CEP131
5	rs10266429	7	71932046	1.39	+	FALSE	CALN1
6	rs17168935	7	15672957	1.34	+	FALSE	MEOX2
7	rs17089043	8	23166281	1.34	+	FALSE	LOXL2
8	rs10853701	18	27355474	1.34	+	FALSE	-
9	rs6998594	8	8848993	1.32	+	FALSE	ERI1,LOC105379227
10	rs650434	6	137889729	1.32	+	FALSE	-
11	rs9539394	13	62632653	1.32	+	FALSE	-
12	rs9888682	15	54173596	1.32	+	FALSE	UNC13C
13	rs11774017	8	98227965	1.32	+	FALSE	LOC101927066
14	rs16976057	15	96612108	1.28	+	FALSE	LOC112268156
15	rs1434302	9	28975706	1.25	+	FALSE	LINGO2
16	rs10985284	9	124270502	1.22	+	TRUE	-
17	rs11141468	9	89249433	1.22	+	FALSE	-
18	rs8070093	17	68474421	1.22	+	TRUE	-
19	rs1864466	2	203856457	1.2	+	FALSE	-
20	rs7523787	1	94330615	1.2	+	FALSE	-
21	rs475385	11	117212892	1.2	+	FALSE	CEP164
22	rs13157029	5	86419364	1.2	+	FALSE	LOC101929380
23	rs1409035	13	67839021	1.16	+	FALSE	LOC105370246
24	rs10129678	14	46059875	1.16	+	FALSE	-
25	rs6915318	6	162254299	1.16	+	FALSE	PRKN
26	rs11873590	18	48100355	1.16	+	TRUE	MAPK4
27	rs6761711	2	65128290	1.16	+	FALSE	-
28	rs1944751	9	18224772	1.16	+	FALSE	ADAMTSL1
29	rs10266429	7	71932046	1.16	+	FALSE	CALN1
30	rs12836163	X	90688866	1.15	+	FALSE	PABPC5-AS1
31	rs11078726	17	7900230	1.15	+	FALSE	-
32	rs332635	6	124749725	1.15	+	FALSE	NKAIN2
33	rs4889210	16	80904237	1.1	+	FALSE	LINC02170
34	rs12578154	12	68177559	1.1	+	FALSE	-
35	rs3913648	17	9597295	1.1	+	FALSE	USP43
36	rs284691	19	34231040	1.1	+	TRUE	CHST8
37	rs604277	18	34972537	1.1	+	FALSE	CELF4
38	rs17100016	14	101669396	1.1	+	TRUE	-
39	rs241166	8	28657998	1.1	+	TRUE	INTS9
40	rs2335524	19	7785698	1.05	+	FALSE	-
41	rs7751879	6	133089557	1.05	+	FALSE	-
42	rs7323755	13	45900267	1.05	+	FALSE	-
43	rs17014512	2	34259759	1.04	+	FALSE	LINC01317
44	rs13317243	3	23185162	1.04	+	FALSE	-
45	rs356612	5	63357558	1.04	+	FALSE	-
46	rs7535074	1	202052416	1.04	+	FALSE	-
47	rs9870623	3	145516298	1.04	+	FALSE	-
48	rs3733103	3	45962595	1.04	+	FALSE	FYCO1
49	rs7864264	9	87639472	1.04	+	FALSE	NTRK2
50	rs443565	15	62788601	1.04	+	FALSE	TLN2

**Table 4:** Top 25 SNP pairs and their associated risk for the lung cancer dataset

Rank	SNP ID	Gene	Recessive	Value	SNP ID	Gene	Recessive	Value	+/− Risk	Risk
1	rs2241873	SAG	FALSE	0	rs2506262	-	FALSE	1	+	3.09
2	rs11649975	NXN	FALSE	0	rs17168935	MEOX2	FALSE	0	+	3.04
3	rs17068439	SYNPR	FALSE	0	rs17168935	MEOX2	FALSE	0	+	3
4	rs7226895	-	FALSE	1	rs17168935	MEOX2	FALSE	0	+	3
5	rs10077754	CTNND2	TRUE	1	rs4889210	LINC02170	FALSE	0	+	3
6	rs11032706	-	FALSE	0	rs16943878	-	FALSE	0	+	2.94
7	rs9318973	-	FALSE	0	rs11708764	-	FALSE	0	+	2.94
8	rs7615876	-	FALSE	0	rs17168935	MEOX2	FALSE	0	+	2.94
9	rs10246303	C1GALT1	TRUE	1	rs16948197	TAOK3	FALSE	0	+	2.94
10	rs10739001	DOCK8	FALSE	0	rs9493858	SGK1	FALSE	0	+	2.89
11	rs9493858	SGK1	TRUE	0	rs9493858	SGK1	FALSE	0	+	2.89
12	rs2151274	-	FALSE	0	rs17168935	MEOX2	FALSE	0	+	2.89
13	rs16914914	-	FALSE	0	rs17168935	MEOX2	FALSE	0	+	2.89
14	rs11649975	NXN	FALSE	0	rs9493858	SGK1	FALSE	0	+	2.89
15	rs4325674	PPP5D1	FALSE	0	rs9517847	CLYBL	FALSE	0	+	2.89
16	rs9678660	-	FALSE	0	rs9493858	SGK1	FALSE	0	+	2.89
17	rs11876308	-	FALSE	0	rs771573	-	FALSE	0	+	2.89
18	rs4455790	RP1L1	FALSE	0	rs2059645	PSD3	FALSE	0	+	2.89
19	rs202589	-	FALSE	0	rs17168935	MEOX2	FALSE	0	+	2.89
20	rs9577032	LOC105370152	FALSE	0	rs6995294	-	FALSE	0	+	2.89
21	rs5982322	MIR325HG	TRUE	0	rs7615876	-	FALSE	0	+	2.89
22	rs2059645	PSD3	TRUE	0	rs4455790	RP1L1	FALSE	0	+	2.89
23	rs11200876	-	FALSE	0	rs12585722	-	FALSE	0	+	2.89
24	rs9595630	N4BP2L2	TRUE	0	rs9493858	SGK1	FALSE	0	+	2.89
25	rs13092498	LINC02008	FALSE	0	rs475385	CEP164	FALSE	0	+	2.89

Now, using FPT to detect significant pairwise SNP interactions, bounding FDR by 5% using the Benjamini-Hochberg procedure, none of the pairs are significant; however, cross validation suggests *T*=1.2, resulting in using 169324 SNP pairs in its analysis. These results suggest although we cannot reliably tell which SNP pairs are true discoveries and which are false discoveries, the information present of the weak SNP pairs outweighs the noise, by aggregating these pairs we can extract the information of encoded in the pairs more than the noise that may be inserted to the decision rule, insert the added information in the prediction rule, and the net effect is more reliable performance. Note that by encoding the SNP pairs in the analysis we may be able to say that for a new test point the net effect of SNP pair interactions is increased risk, i.e., likelihood of being a cancerous point; however, we may not be able to pinpoint which SNP pairs brought us to this conclusion, rather, we can only comment on their agglomerated net effect.

**Fig. 7:**
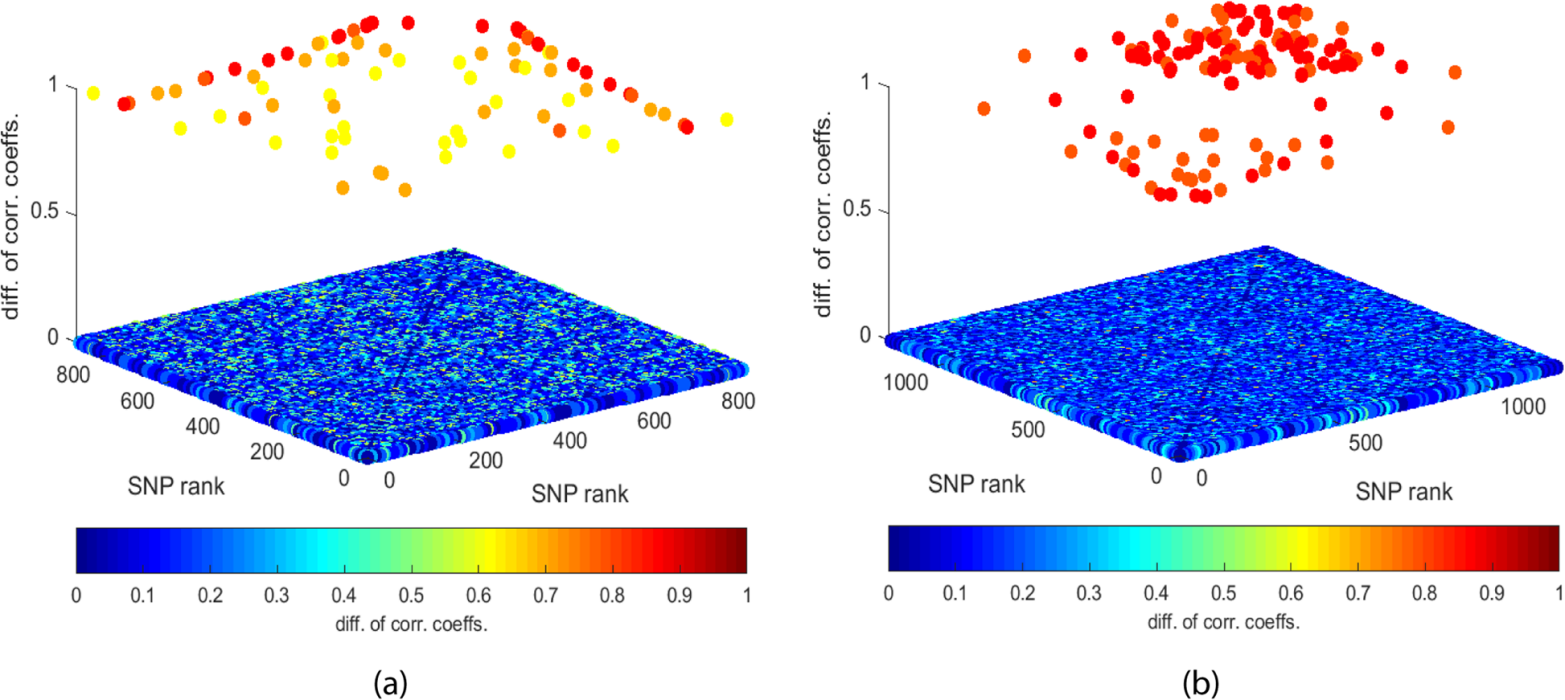
Difference of correlation coefficients for the indicator of both binarized SNPs being present as the interaction pattern for the (a) lung cancer and (b) breast cancer datasets. In the **a** lung cancer and **b** breast cancer datasets only differences larger than 0.5 and 0.8, respectively, are given a non-zero height. The color of each point, denoting a SNP pair, represents the correlation coefficient difference

Finally, we plot the amount of difference in correlation coefficients of SNP pairs in Fig. 7a as we look for the pattern of both binarized SNPs taking value 1 to have a pairwise interaction, i.e., $Z^{f_{i}f_{j}}_{1,1}=1$ being an indicator of an interaction. The x-axis denotes the rank of the first SNP in the pair, and y-axis denotes the second. The z-axis as well as the color of each circle corresponding to a SNP pair denote the amount of the difference in correlation coefficients. To avoid a cluttered figure though, only SNP pairs with differences larger than 0.5 are plotted with a non-zero height. Only a small portion of SNP pairs seem to have large differences in correlation coefficients. Additionally, we observe that many SNPs are common among SNP pairs with large differences in correlation coefficients. In other words, few SNPs are present in many of the SNP pairs with potential interactions. Putting SNP pairs with small differences in correlation coefficients aside, we observe the remaining pairs resemble the pairwise patterns of Fig. 3 describing the heuristic behind LABS-HDMR-CO to merge SNP pairs and construct blocks, suggesting suitability of such strategy might further be biologically motivated.

### Breast cancer

Data obtained in [36] is deposited on GEO with accession number GSE16619, containing dosage data of 42 normal breast tissue samples and 69 cancerous samples. The data is based on the GPL6804 platform measuring 503590 SNPs. Normal and cancerous points comprise classes 0 and 1, respectively. We randomly select 35 normal points and 60 cancerous points for training, use the remaining data for testing, and iterate 100 times. Cross validation sets *D*=1250, *T*_1_=0.25, *T*_2_=1, and *T*_3_=0.5. Linear and quadratic probit with LASSO penalty set *λ* to 0.01 and 0.03, respectively, and the linear probit with elastic net sets *λ*=0.02. Figure 6b plots the ROC curve of different methods. For false positive rates below 5% LABS-HDMR-CO enjoys a higher true positive rate than other algorithms. For higher false positive rates linear probit with elastic net and quadratic problit with LASSO perform almost identical and superior to other algorithms, but are closely followed by LABS-HDMR-CO.

Tables 5 and 6 list the top SNPs and SNP pairs with largest risks used by LABS-HDMR-CO, respectively. We observed some of the SNP IDs present in the data file were not present GPL6804 platform description on the GEO website. For such SNPs we report their ID in the datafile rather than their SNP ID. Although cross validation suggests *D*=1250 for classification, only looking at this set, 269 binarized SNPs are significant using the Fisher’s exact test bounding FDR by 5% using the Benjamini-Hochberg procedure. Going back to the literature we observe several of the genes top SNPs and SNP pairs map to are shown or suggested to be affected in breast cancer. For example, rs13129525 which ranks 9 is located on the FAM171A1 gene which is suggested to be affected in breast cancer [37]. Furthermore, the SNP pair ranking fourth map to CDK19 [38] and CCDC162P [39] genes, which are both shown to be affected in breast cancer. Similar to the lung cancer dataset we again observe that SNP pairs have positive risks, while in contrast to the lung cancer dataset many individual SNPs have negative risk, meaning certain point mutations may reduce the breast cancer relapse risk.

**Table 5:** Several top SNPs and their associated risk for the breast cancer dataset

Rank	SNP ID	Chromosome	Location	Risk	+/− Risk	Recessive	Gene
1	rs17088238	4	58051368	1.83	-	FALSE	-
2	rs9616659	22	48274771	1.7	-	FALSE	C22orf34
3	SNP-A-1916909	–	–	1.67	-	TRUE	-
4	rs1830876	6	51173716	1.67	-	TRUE	-
5	rs1627802	11	15148537	1.61	-	FALSE	INSC
6	rs1514867	5	17797421	1.5	+	TRUE	LOC105374666
7	SNP-A-4276906	–	–	1.5	+	TRUE	-
8	rs10752369	10	15410484	1.49	-	FALSE	FAM171A1
9	rs13129525	4	141069061	1.48	-	TRUE	MAML3
10	rs32489	5	55634181	1.48	-	FALSE	LOC105378977
11	rs2183902	10	74749286	1.48	-	FALSE	CFAP70
12	rs4679029	3	38317718	1.48	-	FALSE	SLC22A14
13	SNP-A-1856501	–	–	1.43	-	FALSE	-
14	rs1459375	10	126998027	1.43	-	FALSE	-
15	rs17450114	5	59517635	1.43	-	FALSE	PDE4D
16	rs10941538	5	17820309	1.39	+	FALSE	LOC105374666
17	rs10192060	2	206075348	1.36	-	TRUE	PARD3B
18	rs10517460	4	37643478	1.34	-	FALSE	TBC1D1
19	rs954765	6	102608467	1.31	+	FALSE	GRIK2
20	rs3867286	11	69483918	1.3	+	TRUE	-
21	rs2069662	5	75952359	1.3	+	FALSE	F2RL2,IQGAP2
22	rs7579373	2	67252044	1.28	+	FALSE	LINC01828
23	rs7205704	16	27308394	1.27	-	FALSE	-
24	rs2274055	13	97925497	1.27	-	FALSE	STK24
25	rs1937991	10	42281298	1.27	-	FALSE	CCNYL2
26	rs17038799	2	36207369	1.27	-	TRUE	-
27	rs2256639	14	68125927	1.27	-	TRUE	RAD51B
28	rs11615811	12	40212891	1.27	-	FALSE	PDZRN4
29	rs17073525	8	5274464	1.26	-	FALSE	-
30	rs719204	20	51997608	1.2	-	FALSE	BCAS1
31	SNP-A-4196610	–	–	1.2	-	FALSE	-
32	rs10741628	11	13851791	1.2	-	TRUE	-
33	rs9820942	3	64535053	1.2	-	TRUE	ADAMTS9
34	rs2063403	2	36082617	1.2	-	TRUE	-
35	rs4413537	5	42681603	1.2	+	TRUE	GHR
36	rs9292856	5	42681023	1.2	+	FALSE	GHR
37	rs7156144	14	67049466	1.19	-	FALSE	TMEM229B,GPHN
38	rs318934	10	131977850	1.16	+	TRUE	LOC107984002
39	rs7607695	2	152607080	1.16	+	TRUE	CACNB4
40	rs7175886	15	31780252	1.16	+	FALSE	RYR3
41	rs433670	16	76173403	1.14	-	FALSE	-
42	rs7278719	21	16607117	1.14	-	FALSE	MIR99AHG
43	rs4716071	6	16541202	1.14	-	FALSE	ATXN1
44	rs6475803	9	2462372	1.14	-	TRUE	LOC101930053
45	rs2322095	18	5439746	1.14	+	TRUE	EPB41L3
46	rs4545261	X	25954300	1.13	+	FALSE	-
47	rs4381121	X	25953702	1.13	+	FALSE	-
48	rs13250548	8	35627942	1.1	-	TRUE	UNC5D
49	rs318931	10	131967734	1.09	+	FALSE	LOC107984002
50	SNP-A-2248670	–	–	1.09	+	TRUE	-

**Table 6:** Top 25 SNP pairs and their associated risk for the breast cancer dataset

Rank	SNP ID	Gene	Recessive	Value	SNP ID	Gene	Recessive	Value	+/− Risk	Risk
1	rs10045084	-	TRUE	0	rs954035	PRRC2C	TRUE	0	+	3.06
2	rs7731058	DOCK2	TRUE	0	rs17022519	LOC102724960	FALSE	0	+	3.06
3	rs7731058	DOCK2	TRUE	0	rs17022501	LOC102724960	FALSE	0	+	3.06
4	rs2817806	CDK19	FALSE	1	rs949881	CCDC162P	FALSE	0	+	3.06
5	rs2715133	GRB10	TRUE	0	rs12878981	TMEM229B,GPHN	FALSE	1	+	3.06
6	rs151130	-	FALSE	1	rs4075386	LRRK1	TRUE	1	+	3.06
7	rs577743	-	FALSE	0	rs7598745	-	TRUE	0	+	3.03
8	rs17829549	-	FALSE	0	rs2322095	EPB41L3	TRUE	0	+	3.03
9	rs11072625	SCAPER	TRUE	0	rs868978	LOC105376137	FALSE	0	+	3.03
10	rs17762161	-	TRUE	1	rs2322095	rs2322095	TRUE	0	+	3.03
11	rs8106386	ZNF420	TRUE	1	rs981013	PDZRN4	FALSE	0	+	3.03
12	rs9384703	CCDC162P	FALSE	0	rs2691184	-	FALSE	1	+	3.03
13	rs6425603	CEP350	FALSE	0	SNP-A-2020595	-	TRUE	1	+	3.03
14	rs1469369	-	TRUE	1	rs2103788	SLC35F4	TRUE	1	+	3.03
15	SNP-A-2041709	-	FALSE	1	SNP-A-4299911	-	TRUE	1	+	3.03
16	rs17006942	-	TRUE	1	rs6460669	GALNT17	FALSE	1	+	3.03
17	SNP-A-1834818	-	FALSE	0	rs590987	NTNG1	TRUE	0	+	3
18	rs11830382	-	FALSE	0	rs1964337	ZNF66	FALSE	0	+	3
19	rs11020107	-	TRUE	1	rs10732488	-	FALSE	0	+	3
20	rs1687064	PRRC2C	FALSE	1	rs10045084	-	TRUE	0	+	3
21	rs10135394	SLC35F4	TRUE	1	rs934034	-	FALSE	0	+	3
22	rs1469369	-	TRUE	1	rs1028458	SLC35F4	FALSE	0	+	3
23	rs4793993	SNF8	FALSE	1	rs5941729	-	FALSE	0	+	3
24	rs2503675	-	FALSE	1	rs1433062	-	FALSE	0	+	3
25	rs2503675	-	FALSE	1	rs10745023	ZNF699	FALSE	0	+	3

Using FPT to detect significant pairwise interactions among the top *D*=1250 SNPs, bounding FDR by 5% using the Benjamini-Hochberg procedure only 4 pairs are significant, although cross validation suggests *T*_2_=1, resulting in using 567190 SNP pairs for classification. This suggests in order to boost our prediction accuracies we need to use many SNP pairs that are not significant, but the information contained in the true discoveries outweighs the noise of the many false discoveries present in the prediction rule. Finally, Fig. 7b plots the differences in correlation coefficients where only pairs with differences larger than 0.8 are assigned a non-zero height. We again observe that few SNPs are common among many pairs with potential interactions.

## Discussion

Analyzing SNP data and developing classification rules given SNP observations is difficult when studying complex diseases. The small-sample high-dimensional nature of the problem, individual SNPs being potentially weak markers, SNPs being categorical variables in nature, and their complex interactions are several important factors that make classifier design a challenging task. Due to each individual SNP contributing only minimally to the class labels, it seems necessary to account for SNP interactions to obtain reliable predictions. The proposed algorithm, LABS-HDMR-CO aims to balance computation cost, complexity, and prediction performance by using a representation that accounts for pairwise interactions. Although higher order HDMR expansions can be considered, given current technology, computation power, and sample sizes, accounting only for pairwise interactions seems to be the most one can hope for.

Interestingly, we observed in our simulated examples described here that the linear models seem to perform better than expected. Although the current simulations are not sufficient to verify the performance and robustness of linear models for SNP classification, we expect this rather good performance to be due to closeness of GLMs to the first degree HDMR expansions. Note that probit and logit links have rather similar graphs, and the linear model of the logit link aims to compute the log likelihood ratio. In other words, the GLM with logit link assumes the log likelihood ratio is linear, and assumes the risk of a SNP with two minor alleles is twice the risk of a SNP with one minor allele. Note the linear term basically resembles the first order HDMR expansion under this additional “dose-effect linearity” assumption. This assumption, similar to the assumptions we made here in the development of LABS-HDMR-CO, reduces complexity and the number of parameters to estimate. Note that the training of a logit model is usually done by maximum likelihood (ML) estimation of the parameters. Finally, note that since probit and logit links are very similar in shape, the superior performance of LABS-HDMR-CO over GLMs may be due to the following three reasons: (1) LABS-HDMR-CO uses second order HDMR expansion while most GLMs used in practice mimic first order HDMR expansion, (2) the preprocessing of LABS-HDMR-CO decomposing SNP dosage data to two indicators seems to better grasp the nonlinear nature of SNPs, while not affecting the flexibility of the algorithm to account for SNPs that are neither recessive nor dominant, and (3) the additional assumptions made in LABS-HDMR-CO seem to enforce less rigidness in the model than the assumptions of GLMs on linear additive risks.

When analyzing cancer datasets reporting SNP dosage values, in the lung cancer dataset we observed that LABS-HDMR-CO may enjoy much superior performance compared with other popular algorithms, and in the breast cancer dataset we observed may perform only slightly inferior to them. Furthermore, when lack of reliable biological knowledge results in the need of considering extremely large number of potential SNP pairs, although we may not be able to reliably detect which SNP pairs are affected in the disease under study, we may be able to aggregate the information of many potential SNP pairs to improve prediction accuracy. In other words, when working with SNP data, it seems sets with large FDRs might still carry enough signal to improve prediction accuracies. Finally, note that the HAPGEN2 data is based on real work sequences, and we can expect it to adequately mimic real world scenarios. We observed much higher accuracies for all classification rules for the HAPGEN2 dataset compared with other cancer datasets. As SNPs seem to be equally weak in all datasets, for instance risks of individual SNPs are not very different, we may hypothesize that the relatively small sample size of cancer datasets may contribute to inferior performance, as the trained classifiers have errors much larger than the Bayes error, and that the larger samples are necessary for more reliable predictions.

## Conclusion

The analysis of genetic variants and their joint effect on complex diseases is a challenging task. In particular, SNPs are a difficult data type to handle due to their high-dimensionality, weak effects of each individual SNP on the phenotype under study, the need to account their joint complex interactions, and their categorical nature. These challenges make it difficult to develop classification rules with reliable predictions, and are exacerbated by the small sample sizes in many applications.

Here we revisited the binary classification problem given categorical SNP observations when reported in dosage, and proposed LABS-HDMR-CO as an algorithm that produces classification rules with good prediction performance that can take several hundred SNPs as input, and account for their pairwise interactions. Additionally, LABS-HDMR-CO is a very fast algorithm in nature, with runtime comparable to a GLM with LASSO penalty. However, due to the 4 parameters to tune (*D*, *T*_1_, *T*_2_, and *T*_3_) in cross validation, the training of the model may become quite computationally expensive. However, note that different thresholds can be evaluated independent of each other, making LABS-HDMR-CO an ideal candidate for parallel computing. Further development of techniques to speed up the training process is an interesting direction for future work that should be explored in more detail.

The categorical nature of SNP dosage data has been a challenge for many machine learning algorithms, and treating them as real numbers has been the focus of many methods. Although the current work requires further study, our initial results suggest that HDMR can a be suitable framework to study SNP data as categorical variables. Additionally, although we only approximate the second order HDMR expansion, we already know that the second order expansion is the best low dimensional representation in the mean square error (MSE) sense [16]. Therefore, it is not surprising that LABS-HDMR-CO enjoys superior prediction performance compared with many algorithms used to study SNP data.

The ability of HDMR to acknowledge categorical nature of SNP data with complex non-linear interactions opens up a new avenue of research to develop low dimensional models suitable for categorical data. Last but not least, the close connection of HDMR with the Sobol indices gives one the ability to identify significant SNP pairs with high interactions, using the same methodology as used for classification problems. Interestingly, we observed that although we may not be able to detect which SNP pairs are affected in the disease under study, the net effect of aggregating many high-profile SNP pairs can boost the prediction accuracy. In other words, in spite of not being able to declare where the “signal” is, i.e., which specific pairwise patterns are affected, it is possible to implicitly extract and take advantage of such information to improve prediction accuracy.

The power, of LABS-HDMR-CO to account for and combine many SNP pairs in its decision rule opens up many avenues of research demanding further investigation. For instance, the type of a classification problem for categorical observations considered here may be extended to non-binary cases (multiple phenotypes) as well, and we will pursue such an extension of our approach in future work. We note, however, that binary classification is the cornerstone of classification theory and many solutions to multiclass problems can be formulated as a sequence of solutions to binary class problems.

## Nomenclature

1_*s*_: indicator function of statement *s* being true *y*: the class label taking values 0 or 1 *D*: the number of features that pass the first phase filtration of the LABS-HDMR-CO classification algorithm *F*: the set of feature indices *u*: an arbitrary subset of *F* *f*_*u*_: the HDMR component for set *u*⊆*F* *X*: the observation random vector comprised of categorical variables *X*_*u*_: restriction of *X* to features in *u*⊆*F* *X*_*f*_: used instead of *X*_{*f*}_ for *f*∈*F* *L*(*X*): the log likelihood ratio of point *X* belonging to class 1 *n*_*y*_: sample size in class *y*${\hat {p}^{f}_{c},{y}}$: OBF’s estimate of the probability of feature *f* taking value *c* in class *y*${\hat {p}^{f_{i},f_{j}}_{c_{i},c_{j}}(y)}$: OBF’s estimate of the probability of features *f*_*i*_ and *f*_*j*_ taking values *c*_*i*_ and *c*_*j*_, respectively, in class *y*${q^{c}_{u}}$: the HDMR coefficient of when feature set *u* takes value *c* *C*_*f*_: the set of categorical values feature *f*∈*F* can take *r*^*f*^: the risk associated to binarized feature *f*${r^{f}_if_{j}}$: the risk associated to binarized feature pair comprised of *f*_*i*_ and *f*_*j*_ *R*(*X*): the risk associated to observation *X*${\mathscr {S}}$: the training sample${\mathscr {S}_{y}}$: the training sample in class *y* *S*(*u*): the Sobol index of feature set *u* *S*^*c*^(*u*): the main effect Sobol index of feature set *u* *T*: the threshold used to assign a class to a test point *T*_1_: the threhsold used to remove weak features *T*_2_: the threhsold used to remove weak feature pairs *T*_3_: the threhsold used to remove weak feature blocks *b*^∗^: the estimated approximate HDMR coefficients obtained from the training sample${w^{q}_{u}}$: the HDMR coefficient of when the observed values of feature set *u* satisfy constraint *q* *Z*=*f*(*X*): is the dependent varibale whose HDMR expansion is being computed *E*_*d*_(*Z*|*X*): the *d*^*th*^ order HDMR expansion of *Z* given observation *X*${Z^{f_{i},f_{j}}_{c_{i},c_{j}}}$: the indicator of features *f*_*i*_ and *f*_*j*_ taking values *c*_*i*_ and *c*_*j*_, respectively${\hat {\rho }^{f_{i},f_{j}}_{y}}$: correlation coefficient between *f*_*i*_ and *f*_*j*_${k_{c_{i} c_{j}}^{f_{i},f_{j}}(y)}$: the ratio between probability mass function value of observing features *f*_*i*_ and *f*_*j*_ taking values *c*_*i*_ and *c*_*j*_,repectievly, and the probability mass function value assuming *f*_*i*_ and *f*_*j*_ are independent in class *y*${z^{f_{i},f_{j}}_{y}}$: Fisher’s r to z transform value for Bernoulli random variables *f*_*i*_ and *f*_*j*_${N^{f_{i}}_{c_{1} c2}}$: the block comprised of feature pairs that (a) have large negative risks, (b) contain *f*_*i*_, (c) *f*_*i*_ takes value *c*_1_, and (d) the other feature in the pair, *f*_*j*_, takes value *c*_2_${P^{f_{i}}_{c_{1} c2}}$: the block comprised of feature pairs that (a) have large positive risks, (b) contain *f*_*i*_, (c) *f*_*i*_ takes value *c*_1_, and (d) the other feature in the pair, *f*_*j*_, takes value *c*_2_ *r*^*A*^: the risk associated to block *A* being the ratio of observed pairwise feature patterns of *A* averaged over all samples

## Supplementary information

**Additional file 1** Supplementary: top SNPs of LABS-HDMR-CO. The top SNPs used by LABS-HDMR-CO for the HAPGEN2, breast cancer, and lung cancer datasets are provided in the Supplementary.

## Data Availability

A MATLAB implementation of LABS-HDMR-CO is available on Github. GWAS datasets are publicly available on GEO, and the synthetically generated HAPGEN2 data is available upon request.

## Abbreviations

AUC: area under curve
DNA: deoxyribonucleic acid
FDR: false discovery rate
FPT: fixed pattern test
GEO: gene expression omnibus
GLM: generalized linear model
HDMR: high dimensional model representation
LABS-HDMR-CO: linear approximation for block second order HDMR expansion for categorical observations
LASSO: least absolute shrinkage and selection operator
MSE: mean square error
ROC: receiver operator characteristic
SNP: single nucleotide polymorphism
